# PD-L1 enhances migration and invasion of trophoblasts by upregulating ARHGDIB via transcription factor PU.1

**DOI:** 10.1038/s41420-022-01171-6

**Published:** 2022-09-22

**Authors:** Ruonan Zhang, Linyan Jia, Lulu Meng, Hao Peng, Donghai Zhang, Qizhi He, Tao Duan, Kai Wang

**Affiliations:** 1 Clinical and Translational Research Center, Shanghai Key Laboratory of Maternal Fetal Medicine, Shanghai Institute of Maternal-Fetal Medicine and Gynecologic Oncology, Shanghai First Maternity and Infant Hospital, School of Medicine, Tongji University, Shanghai, P. R. China; 2 Department of Pathology, Shanghai Key Laboratory of Maternal Fetal Medicine, Shanghai Institute of Maternal-Fetal Medicine and Gynecologic Oncology, Shanghai First Maternity and Infant Hospital, School of Medicine, Tongji University, Shanghai, P. R. China; 3 Department of Obstetrics, Shanghai Key Laboratory of Maternal Fetal Medicine, Shanghai Institute of Maternal-Fetal Medicine and Gynecologic Oncology, Shanghai First Maternity and Infant Hospital, School of Medicine, Tongji University, Shanghai, P. R. China

**Keywords:** Diseases, Disease model, Reproductive disorders

## Abstract

As the main constituent cells of the human placenta, trophoblasts proliferate, differentiate, and invade the uterine endometrium via a series of processes, which are regulated exquisitely through intercellular signaling mediated by hormones, cytokines, and growth factors. Programmed cell death ligand 1 (PD-L1) is a biomarker of the response to immune checkpoint inhibitors and can regulate maternal-fetal immune tolerance during pregnancy progression. Recently, it was found that PD-L1 may regulate obstetric complications by affecting the function of trophoblasts. Therefore, we examined the expression and localization of PD-L1 in the human placenta and observed the effects of PD-L1 on trophoblasts migration and invasion in both the trophoblasts line HTR-8/SVneo and an extravillous explant culture model. Finally, we explored the molecular mechanisms underlying PD-L1-regulated trophoblasts migration and invasion through RNA sequencing and bioinformatics analysis. Our data showed that PD-L1 was mainly expressed in syncytiotrophoblasts and that its protein levels increased with gestational age. Interestingly, the protein expression of PD-L1 was significantly decreased in placentas from pregnancies with preeclampsia compared with normal placentas. Importantly, the migration and invasion abilities of trophoblasts were significantly changed after knockdown or overexpression of PD-L1 in HTR-8/SVneo cells and an extravillous explant culture model, which was partially mediated through the transcription factor PU.1 (encoded by Spi1)-regulated Rho GDP-dissociation inhibitor beta (ARHGDIB) expression. These results suggested that PD-L1 was highly involved in the regulation of trophoblasts migration and invasion, providing a potential target for the diagnosis and treatment of placenta-derived pregnancy disorders.

## Introduction

The placenta is a transient organ mediating nutrient and gas exchange between the mother and fetus. Dysfunction of the placenta is considered to elevate the risk of pregnancy complications [1]. Trophoblasts are the main constituent cells of the human placenta, and the migration and invasion of trophoblasts are necessary for the vascular remodeling process. The dysfunction of trophoblasts is closely related to the occurrence of placental diseases, such as preeclampsia (PE) [2]. Recent studies on the trophoblasts migration process have mainly focused on the transformation of the trophoblasts invasion phenotype, reduction in extracellular matrix adhesion, and factors promoting cellular migration [3–5].

Programmed cell death ligand 1 (PD-L1), also known as B7-H1/CD274, is a member of the B7 family of immune molecules and is involved in the regulation of cellular and humoral immune responses. It was first synthesized from a human placental cDNA library in 1999 and is highly expressed in the placenta compared with other normal tissues [6, 7]. Current studies have primarily concentrated on the role of PD-L1 in tumor immune escape as an immune checkpoint, as well as maternal-fetal immune tolerance during pregnancy. Recently, it was reported that PD-L1 is also deeply involved in the regulation of tumor cell migration and invasion [8, 9]. Interestingly, the process of placentation bears several striking similarities to tumor cell metastasis, and both trophoblasts and tumor cells express immunosuppressive molecules, including HLA-G and PD-L1 [10]. At the maternal-fetal interface, several study groups have assessed whether PD-1 is expressed on maternal immune cells, especially activated T cells in the decidua. However, the translocation and function of PD-L1 in trophoblasts is still unclear [11–13].

In this study, we examined the expression and localization of PD-L1 in the human placenta and observed the effects of PD-L1 on trophoblasts migration and invasion in both the trophoblasts line HTR-8/SVneo and a human extravillous explant culture model. Finally, we explored the molecular mechanisms underlying PD-L1-regulated trophoblasts migration and invasion through RNA sequencing and bioinformatics analysis. This study enables us to further understand the pathogenesis of placental disorders by dysfunction of trophoblasts.

## Materials and methods

### Study population and sample collection

All participants in the study were pregnant women who underwent natural delivery or induced abortion in Shanghai First Maternity and Infant Hospital from May 2019 to May 2021. The patients in this study were divided into early pregnancy (*n* = 9, 6–8 weeks of gestation, 25–35 years of aged), middle pregnancy (*n* = 4, 13–15 weeks of gestation, 25–35 years of aged), normal late pregnancy (*n* = 11, 37–39 weeks of gestation, 29–35 years of aged), and preeclampsia (*n* = 6, 33–39 weeks of gestation, 28–35 years of aged), as shown in Table 1. In this study, the placental tissues of all patients were obtained from the operating room with the approval of the Scientific Ethics Committee of Shanghai First Maternity and Infants Hospital, affiliated with Tongji University. After the blood clots were cleaned on ice with PBS, they were cut into 1 cm × 1 cm tissue blocks and stored in liquid nitrogen in frozen storage tubes for further use.

**Table 1:** The characteristics of women from normal and preeclampsia pregnancies.

Characteristics	Normal (*n* = 6)	Preeclampsia (*n* = 6)	*P* value (Mann–Whitney *U*-test)
Maternal age (yr)	32.33 ± 1.37	31.33 ± 2.80	NS
Gestational age (wk)	38.57 ± 0.72	36.02 ± 2.51	NS
Gravidity	1.83 ± 0.41	1.67 ± 1.21	NS
Parity	0.50 ± 0.55	0.17 ± 0.41	NS
Systolic blood pressure (mmHg)	115.00 ± 6.99	134.83 ± 27.63	0.48
Diastolic blood pressure (mmHg)	73.67 ± 6.62	90.33 ± 22.16	0.07
Proteinuria (g/24h)	—	0.30 ± 1.7	—
Uric acid (μg/l)	238.83 ± 37.74	384.17 ± 72.96	<0.05
Creatinine (μg/l)	42.83 ± 6.62	55.33 ± 12.27	<0.05

Data were expressed as means ± SEM, *p* ≤ 0.05 is considered statistically significant.

### Western blot analysis

The concentration of proteins was quantified using the Pierce BCA Protein Assay Kit (Thermo Fisher Scientific, Waltham, MA, USA), following the manufacturer’s instructions. Proteins were separated by 10% SDS PAGE gels and transferred to PVDF membranes by gel electrophoresis and electroblotting. After blocking with 5% BSA, the blots were probed with primary antibodies at 4 °C overnight. Then, the membranes were washed and incubated with secondary antibodies. Ultimately, proteins were visualized using enhanced chemiluminescence reagents (Thermo Fisher Scientific). The antibodies used are listed in Table S1. The relative protein expression levels were analyzed by densitometry using ImageJ imaging analysis software (NIH).

### Immunofluorescence staining

Cell suspensions were seeded on the central concavity of a special 35-mm glass-bottom plate (Nest, China) at 50% confluence. After three washes with ice-cold PBS, the cells were fixed with 4% paraformaldehyde (PFA, Dingguo, China) for 15 min and then permeabilized with 0.1% Triton X-100 (Dingguo) for 10 min. Subsequently, the cells were blocked with 5% bovine serum albumin (Thermo Fisher Scientific) for 1 h at room temperature and incubated with an appropriate concentration of primary antibodies against PD-L1 (1:500; Abcam, London, UK) overnight at 4 °C. Then, the cells were incubated with Alexa Fluor® 488-conjugated goat anti-rabbit IgG (1:200, Abcam) or goat anti-mouse IgG (1:200, Abcam) secondary antibodies for 1 h. The stained cells were observed under a confocal (Leica TSC SP8, Mannheim, Germany). The antibodies used are listed in Table S2.

### RNA isolation and quantitative RT‐PCR

Total RNA was extracted using TRIzol (Thermo Fisher Scientific), and RNA was then reverse transcribed using the SuperScript First Strand cDNA System (Takara) according to the manufacturer’s instructions. Quantitative RT-PCR (qRT-PCR) was performed using the SYBR Green PCR master mix (Takara) and the StepOnePlus PCR system (Thermo Fisher Scientific) according to the manufacturer’s instructions. The housekeeping gene GAPDH was used as an endogenous control. The primer sequences are shown in Table S3.

### Immunohistochemical

Placental tissues were immersion-fixed in 4% buffered formalin and then transferred to paraffin. Tissue sections of 3–5 μM in thickness were cut from the paraffin-embedded tissues, mounted on poly-l-lysine-coated slides, deparaffinized in xylene, dehydrated in alcohol and then stained with H&E. Some sections were stained for PD-L1 (1:50; Cell Signaling Technology, Danvers, MA, USA) using the streptavidin-biotin-horseradish peroxidase complex formation method.

### Cell culture

HTR-8/SVneo and 293 T cells obtained from ATCC (Rockville, MD, USA) were maintained at 37 °C in a humidified atmosphere with 5% CO_2_ and 95% air. Cells were cultured in DMEM/F12 medium (HyClone, Logan, UT, USA) or DMEM-high glucose medium (HyClone) with 10% fetal bovine serum (FSB, Gibco) and 1% penicillin/streptomycin.

### Cell proliferation assay

To test the effect of PD-L1 on HTR-8/SVneo cell proliferation, 3 × 10^3^ cells per well were plated in 96-well culture plates in 100 μL of DMEM/F12 medium. The medium was changed to one that contained 1% FBS with fresh medium replaced at 16 h. After 48 h, relative cell numbers were determined using MTS (Promega, Madison, WI, USA) reagent in a 96-well plate reader at 490 nm.

### Cell migration and invasion assay

The migration of HTR-8/SVneo cells was determined using a modified system according to the manufacturer’s instructions (BD Bioscience, Heidelberg, Germany). In this assay, cell migration was monitored using a 24-well transwell plate with inserts containing 8 μm pores (Costar, Corning, NY, USA). In brief, 5 × 10^4^ cells were seeded on the upper side of the transwell inserts in a serum-free medium. DMEM/F12 containing 1% FBS was added to the companion plate. Following incubation at 37 °C with 5% CO_2_ and 95% air for 16 h, the fluorescent stain calcein-AM (Thermo Fisher Scientific) was added to each chamber and incubated for 30 min. The numbers of migrated cells were determined by fluorescence image analysis (Nikon TI-S, Tokyo, Japan).

The capacity of HTR-8/SVneo cells to invade Matrigel was determined using 24-well transwell plates. In this assay, the upper chamber of the insert was precoated with 100 μL of a 1:20 dilution of Matrigel (BD Bioscience) in a standard medium for 60 min at 37 °C. After hydration with PBS for 60 minutes, 7 × 10^4^ cells were seeded into the upper side of the transwell inserts in a serum-free medium. DMEM/F12 containing 1% FBS was added to the companion plate. Following incubation at 37 °C with 5% CO_2_ and 95% air for 16 h, calcein-AM (Thermo Fisher Scientific) was added to each chamber and incubated for 30 min. The numbers of migrated cells were determined by fluorescence image analysis.

### Dual-luciferase reporter assay

HTR-8/SVneo cells were transfected with 100 ng of GM-4629 construct that carries the wildtype or mutant type (ARHGDIB-WT or ARHGDIB-MUT, respectively) and 100 ng of PGMLV-6395 overexpressing PU.1 or scramble control (NC), together with 10 ng of pRL-TK control vector (encoding Renilla luciferase). The above vectors were purchased from Shanghai Genomeditech (Shanghai, China) and verified by DNA sequencing. Thirty-six hours after transfection, the activities of firefly and Renilla luciferase were measured using a Dual-Glo Luciferase Assay System (Promega) and a GloMax 96 microplate luminometer (Promega). Firefly luciferase activity was normalized to Renilla luciferase activity.

### Transfection

HTR-8/SVneo cells were transfected with lentivirus carrying PD-L1 (Lenti-PD-L1) or green fluorescent protein (Lenti-Ctrl). Briefly, subconfluent (50%) HTR-8/SVneo cells were precultured with DMEM/F12 (no FBS or penicillin/streptomycin) for 2 h. Then, HTR-8/SVneo cells with a predetermined multiplicity of infection (MOI) of Lenti-PD-L1 or Lenti-Ctrl in a complete DMEM/F12 medium were cultured for up to 2 days. Then, 1.0 μg/ml puromycin was used to filter the cells that were successfully transfected.

Small interfering RNA (siRNA) targeting PD-L1 was designed based on the human PD-L1 coding sequence (Gene ID: 29126) and synthesized by RIBOBIO (Guangzhou, China). Briefly, HTR-8/SVneo cells were inoculated in one well of a six-well plate seed. When the cell density reached 50%, the complete medium was removed, and serum-free DMEM/F12 medium was added for 1 h. A concentration of 50 nM oligomers (siCtrl or siPD-L1 group) was mixed with Lipofectamine 2000 (Thermo Fisher Scientific), diluted in serum-free DMEM/F12 medium, and incubated for 15 min at room temperature. Then, this mixture was added to the cells. After 6 h, the medium was removed and washed twice with PBS, and the fresh complete medium was replaced. After 48 h, RNA and protein were collected. The concentrations of siRNA targeting ARHGDIB (Gene ID: 397) and SPI1 (Gene ID: 6688) were both 100 nM.

### First-trimester human placental explant culture

Briefly, villous explants with potential EVT columns were carefully dissected and positioned on 24-well plates (Corning) precoated with 200 μL of Matrigel (BD Biocoat, 356234), which was diluted 1:1 with DMEM/F12 medium. After 24 h of culture, villous tips were examined under a dissecting microscope for successful EVT outgrowth. All successful explants were selected for treatment with 250 nM of oligomers (siCtrl or siPD-L1 group). Explants were photographed immediately after adding the treatment and subsequently at 48 h using a Leica DFC400 camera attached to a dissecting microscope. ImageJ was used to measure the distance of EVT outgrowth. Specifically, the total outgrowth area was calculated by subtracting the distance at the end point from the initial area upon treatment. Each experiment was designed with a minimum of four replicates and was repeated on three different placentas. Then, the expression of HLA-G (Abcam), a marker protein of EVT, was detected by immunofluorescence staining. Additionally, the fluorescent microscope (Leica DFC400) was used to demonstrate the efficient knockdown of PD-L1.

### Statistical analyses

Data were expressed as the means ± SD and were analyzed using GraphPad Prism 8 (GraphPad Software Inc., San Diego, CA, USA). Statistical significance was analyzed by unpaired Student’s *t*-tests or one-way ANOVA. A *p* value < 0.05 was considered statistically significant.

## Results

### Expression and distribution of PD-L1 in human placental tissues

**Fig. 1:**
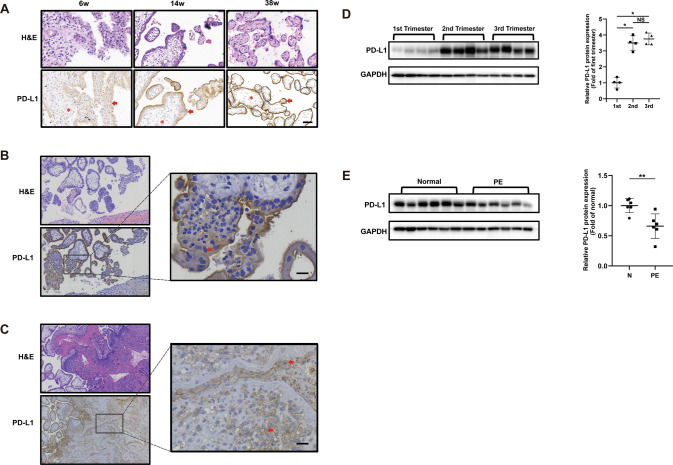
Expression and distribution of PD-L1 in placental tissue. HE staining and immunolocalization of PD-L1 in human placental villi from women with pregnancies (**A** Bar, 100 μm), EVTs in placental villi (**B** Bar, 10 μm), and invasive EVTs in maternal endometrium (**C** Bar, 20 μm). The brown color indicates positive staining for PD-L1. Arrowheads, trophoblasts; asterisks, lumens of blood vessels. Western blot analysis for PD-L1 in human placentas from different trimesters (**D**) and preeclampsia pregnancies (**E**). Representative Western blot images are shown for PD-L1 and GAPDH. *p < 0.05 indicates that the difference between the two groups was statistically significant.

To explore the functional role of PD-L1 in placental trophoblasts, we first determined the location and expression of PD-L1 protein in the placenta by immunohistochemistry and Western blotting. Our data showed that PD-L1 was mainly expressed in the villous syncytiotrophoblast layer of placental villi throughout gestation (Fig. 1A). Notably, PD-L1 is abundantly expressed in migratory extravillous trophoblasts (EVTs) of the distal anchoring column (Fig. 1B) and invasive EVTs in the maternal endometrium (Fig. 1C). The protein levels of PD-L1 increased with gestational age, as shown by Western blot detection. Moreover, high levels of PD-L1 were observed in placental tissues from both the second and third trimesters (Fig. 1D). Interestingly, the placenta of preeclampsia patients contained lower protein levels of PD-Ll than that from healthy pregnancies (Fig. 1E), which was consistent with results described in other reports [10, 14]. Together, these findings indicate that the PD-L1 protein is deeply involved in the regulation of trophoblasts functions, especially EVT cell migration and invasion.

### Effect of PD-L1 on the migration and invasion of trophoblasts in vitro and ex vivo

**Fig. 2:**
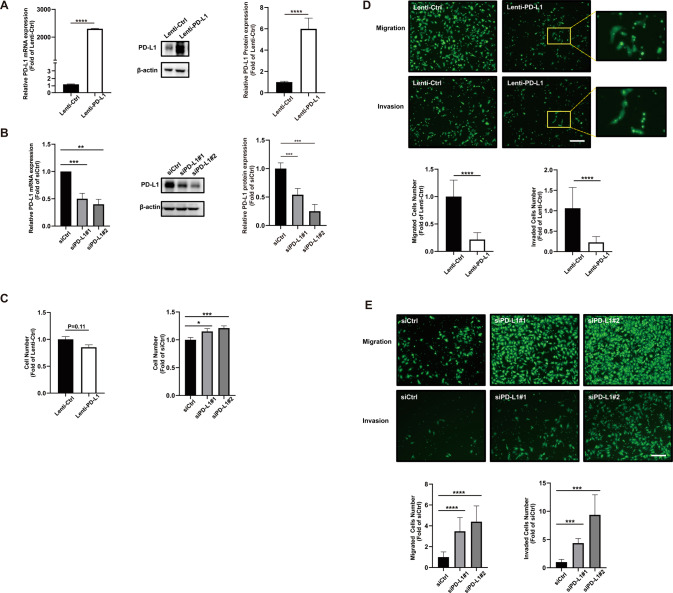
Effects of changes in PD-L1 expression on trophoblast function. **A**, **B** The mRNA and protein levels of PD-L1 demonstrate the efficient overexpression or knockdown of PD-L1 in HTR-8/SVneo cells. Effects of PD-L1 overexpression or knockdown on the proliferation (**C**), migration (**D**), and invasion (**E**) of HTR-8/SVneo cells. Each hole had four fields of view, and the number of cells in the field of view was calculated. The cell number is relative to three independent experiments. (****P < 0.0001) Bar, 200 μm.

The migration and invasion abilities are features of trophoblasts function in placental development during pregnancy. To observe the effects of PD-L1 on trophoblasts migration and invasion, we downregulated and stably overexpressed PD-L1 in the HTR-8/SVneo cell line via siRNA transfection and lentivirus infection (Fig. 2A, B), respectively. As expected, knockdown of PD-L1 led to a significant increase in both the migration and invasion of HTR-8/SVneo cells. However, upregulation of PD-L1 resulted in a decrease in HTR-8/SVneo cellular functions. Additionally, PD-L1-specific siRNA slightly enhanced HTR-8/SVneo cell proliferation (Fig. 2C–E).

**Fig. 3:**
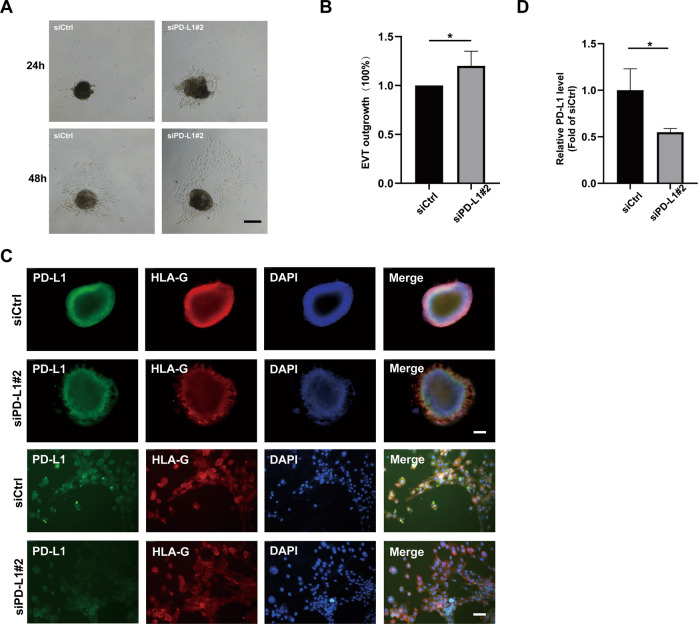
PD-L1 reduced trophoblasts outgrowth in extravillous explant cultures. **A** Extravillous explants were maintained in culture on Matrigel. Bar, 200 μm. **B** Statistical assay of the migration distance of villous tips (%). **C** Immunofluorescence staining showed an obvious decrease in PD-L1 after 60 h, and the level of PD-L1 expression was assessed using confocal microscopy (**D**). Green fluorescence signals indicate bound anti-PD-L1 antibodies; HLA-G staining is visualized as red; and a DAPI-stained nucleus is blue. Bar, 100 μm of top two rows and 25 μm behind two rows.

To further test the role of PD-L1 in trophoblasts migration and invasion, we created an ex vivo placental villi explant culture model in which human placental explants from the first trimester were cultured on Matrigel and treated with either control siRNA (siCtrl) or PD-L1-specific siRNA (siPD-L1). Our data showed that siPD-L1 significantly enhanced explant outgrowth after 48 h of treatment when compared with siCtrl (Fig. 3A, B). Moreover, whole-mount immunofluorescence staining showed that trophoblasts that migrated out of the villi were positive for both HLA-G and PD-L1, and a robust knockdown efficiency of PD-L1 in the siPD-L1 group was observed (Fig. 3C, D). Collectively, these results suggested that siRNA-mediated PD-L1 knockdown in early villi explants significantly suppressed the outgrowth (migration and invasion) of EVTs.

### The effect of PD-L1 on downstream gene expression was analyzed by RNA sequencing

**Fig. 4:**
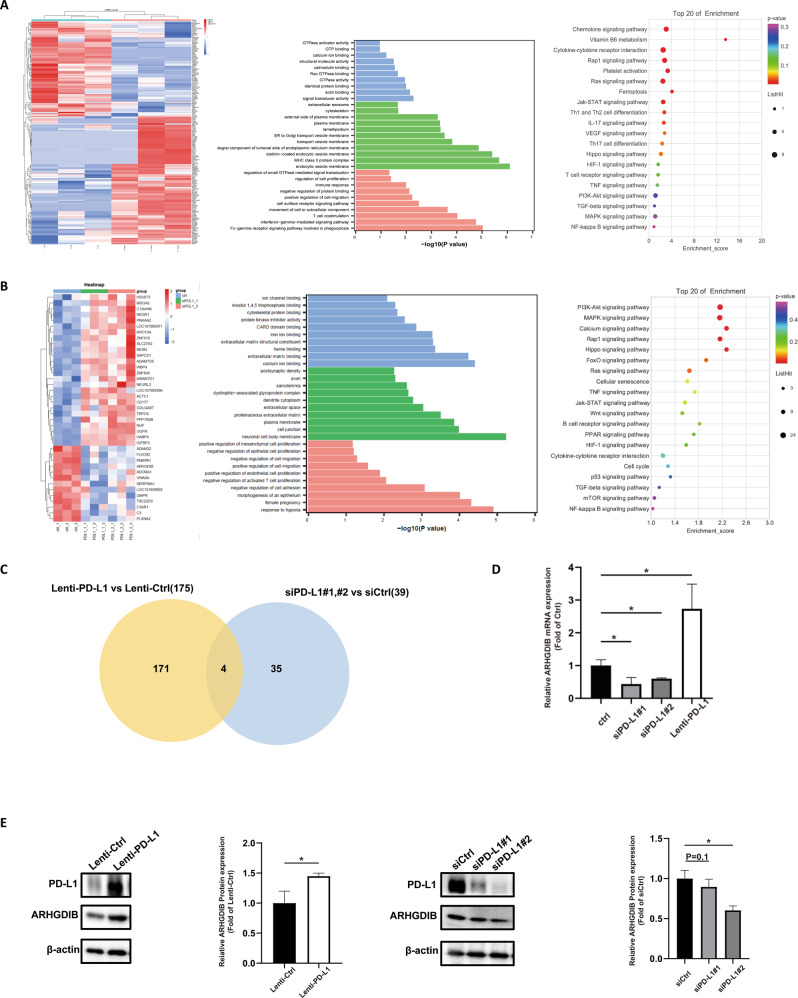
The effect of PD-L1 on downstream gene expression was analyzed by RNA sequencing. **A**, **B** A heatmap was constructed from the genes that demonstrated differential expression between the PD-L1 group and the control group. The biological processes, cellular components, and molecular functions in which the most significantly upregulated or downregulated genes were enriched by GO analysis. Twenty pathways were significantly enriched by KEGG. **C** Venn diagram of the comparison of significantly upregulated or downregulated genes among PD-L1 siRNA and PD-L1 overexpression samples. The mRNA (**D**) and protein (**E**) levels of ARHGDIB, the target gene of PD-L1 identified by RNA-seq.

To investigate the internal mechanism by which PD-L1 affects trophoblasts migration and invasion, we investigated candidate downstream genes by performing RNA-sequencing analysis in both Lenti-PD-L1-infected and siPD-L1-transfected HTR-8/SVneo cells. There were 175 and 39 differentially expressed genes (DEGs) in these two groups in comparison with their corresponding controls, respectively (adjusted *p* value of less than 0.05 and an absolute log fold change of greater than 0.5 or 1.5-fold change). Meanwhile, functional clustering by GO analysis revealed that the DEGs from these two groups were mainly associated with the regulation of cell migration and proliferation at the molecular function level (Fig. 4A–C). Across the two comparisons, the DEGs Rho GDP-dissociation inhibitor beta (ARHGDIB), Serpin Family A Member 1 (SERPINA1), Plexina2 (PLXNA), and LOC107985971 were significantly upregulated in all PD-L1 overexpression samples but downregulated in all PD-L1 knockdown samples (Table 2). Subsequent validation of the RNA-seq results using RT–qPCR showed that the expression level changes of all 3 genes were consistent with the RNA-seq results (Supplementary Fig. 1). Among these three characterized genes, ARHGDIB was considered to be closely involved in the negative regulation of trophoblasts migration. We also demonstrated that its expression was increased at both the mRNA and protein levels in overexpression samples and was decreased at the mRNA levels in knockdown samples (Fig. 4D, E).

**Table 2:** The most significant genes (4) were enriched by the degree of deviation and molecular function. Information (*p* value, product, and function) about differentially expressed genes in HTR-8/SVneo knockdown vs. control and HTR-8/SVneo overexpression vs control cells.

	*P* value (siPD-L1#1 vs siCtrl)	*P* value (siPD-L1#2 vs siCtrl)	*P* value (Lenti-PD-L1 vs Lenti-Ctrl)	Product	Function
SERPINA1	0.002164885	0.026318736	0.021957559	serpin family A member 1	Complement and coagulation cascades
PLXNA2	9.27E-08	2.45E-05	1.65E-12	plexin A2	Somitogenesis branchiomotor neuron axon guidance|regulation of cell migration|regulation of axon extension involved in axon guidance
ARHGDIB	3.08E-10	0.00018831	2.62E-10	Rho GDP-dissociation inhibitor beta	Rho GDP-dissociation inhibitor cytosol|movement of cell or subcellular component|negative regulation of cell adhesion|regulation of small GTPase mediated signal |negative regulation of trophoblast cell migration
LOC107985971	0.000751314	0.007920615	0.007920615	uncharacterized	

### Effect of ARHGDIB on the proliferation, migration, and invasion of trophoblasts

**Fig. 5:**
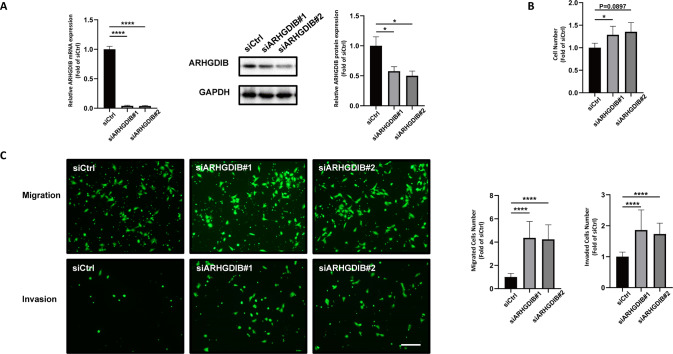
Effect of ARHGDIB on the proliferation, migration, and invasion of trophoblasts. **A** The mRNA and protein levels of ARHGDIB were detected by RT–qPCR and western blot, respectively, to demonstrate the efficient knockdown of ARHGDIB in HTR-8/SVneo cells. Effects of ARHGDIB knockdown on the proliferation (**B**), migration and invasion (**C**) of HTR-8/SVneo cells. Each hole had four fields of view, and the number of cells in the field of view was calculated. The cell number is relative to three independent experiments. (****P < 0.0001) Bar, 200 μm.

To further observe the effects of ARHGDIB on the migration and invasion of trophoblasts, we downregulated ARHGDIB expression by using specific siRNA. Both the mRNA and protein levels of ARHGDIB were robustly reduced following ARHGDIB siRNA treatment (Fig. 5A). Loss of ARHGDIB significantly increased the proliferation, migration, and invasion of HTR-8/SVneo cells (Fig. 5B, C).

### PD-L1 transcriptionally upregulated ARHGDIB through the transcription factor PU.1

**Fig. 6:**
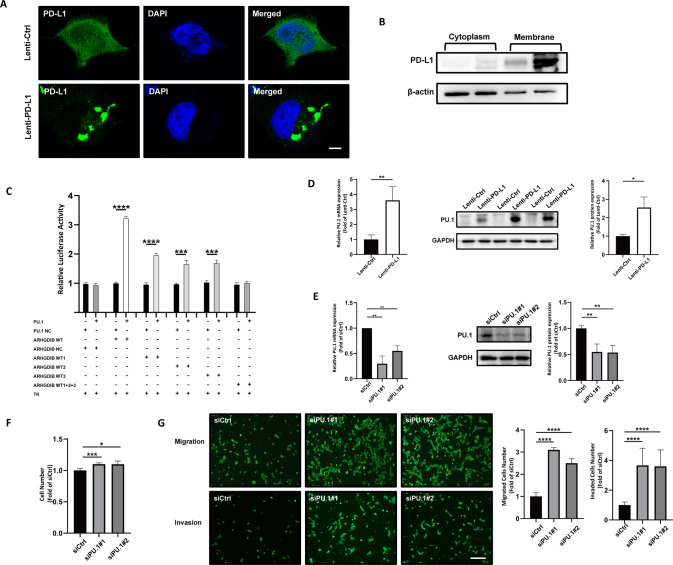
PD-L1 transcriptionally upregulated ARHGDIB through the transcription factor PU.1. **A**, **B** The expression of PD-L1 did not affect its localization in cells. Green indicates positive staining for PD-L1, and blue indicates DAPI. Bar, 5 μm. **C** Luciferase reporter assay showed that PU.1 bound to sites 1, 2, and 3 on the ARHGDIB promoter. **D** The mRNA and protein levels of PU.1 (SPI1) in PD-L1-overexpressing or control HTR-8/SVneo cells. **E**–**G** Knockdown of PU.1 promoted HTR-8/SVneo cell proliferation, migration, and invasion.

To determine how PD-L1 affects the expression of ARHGDIB, we first observed PD-L1 translocation after PD-L1 overexpression in HTR-8/SVneo cells by confocal detection (Fig. 6A) and Western blotting (Fig. 6B). We found that PD-L1 was mostly expressed in the cytoplasm and was not changed after overexpression of PD-L1 in HTR-8/SVneo cells. Based on our previous RNA-sequencing analysis, the E26 transformation-specific family (ETS) and P53 were highly associated with DEGs (Supplementary Fig. 2). The majority of these DEGs were largely regulated by the ETS (E26 transformation-specific family) family, especially PU.1, a well-known transcription factor. By applying the JASPAR tool (http://jaspar.genereg.net/), we confirmed the DNA motif and identified 3 binding sites on the *ARHGDIB* promoter, as shown in Supplementary Fig. 3. Double luciferase reporter gene experiments demonstrated the direct correlation between the transcription factor PU.1 and the ARHGDIB gene promoter, and three region sites (-1967 to −1979, −649 to −666, −8 to −22) of the ARHGDIB promoter were further confirmed to have binding activity with this transcription factor (Fig. 6C) by mutating these binding sites. In addition, our data also showed that the mRNA and protein levels of PU.1 were significantly elevated after PD-L1 overexpression in HTR-8/SVneo cells (Fig. 6D), which indicated that PD-L1 likely regulated ARHGDIB expression via upregulation of PU.1. Finally, we examined the proliferation, migration and invasion ability of trophoblasts after PU.1 knockdown (Fig. 6E). As expected, the proliferation, migration and invasion abilities of HTR-8/SVneo cells were significantly upregulated after PU.1 knockdown (Fig. 6F, G). This is in agreement with our hypothesis that PD-L1 is transcriptionally upregulated by upregulation of the transcription factor PU.1.

## Discussion

It is well known that the imbalance of PD-1/PD-L1 can lead to severe pregnancy complications by contributing to the maintenance of immune tolerance, including PE [15–18]. To date, little is known about the role of PD-L1 in trophoblasts functions during pregnancy except for that of immune regulation. However, few articles have reported widespread expression of PD-L1 in trophoblast-related malignant diseases [19].

In this study, we observed that PD-L1 was mainly expressed in the syncytiotrophoblast of placental villi and that its expression increased with gestational age. This finding is in agreement with other reports [13, 14]. Importantly, we found that PD-L1 was highly expressed in EVTs, which migrated from the placental villi and partially invaded the maternal decidua, suggesting that PD-L1 was closely correlated with trophoblasts invasion function. Indeed, our present data showed that the migration and invasion of trophoblasts were significantly changed after the alteration of PD-L1 protein levels in HTR-8/SVneo cells, a widely used EVT cell line. PD-L1 expression was negatively correlated with these functions. These results are fully supported by another study showing that reduced PD-L1 expression could attenuate HTR-8/SVneo trophoblasts cell invasion [20].

To explore the molecular mechanism by which PD-L1 affects trophoblasts function, we performed RNA sequencing using both PD-L1 knockdown and PD-L1-overexpressing HTR-8/SVneo cells. We demonstrated that ARHGDIB, a family of Rho guanosine diphosphate dissociation inhibitors (RhoGDIs), is closely linked with PD-L1 expression. Further experiments demonstrated that ARHGDIB also negatively regulated trophoblasts migration and invasion. Moreover, ARHGDIB-specific siRNA completely diminished PD-L1-inhibited cell migration, which was consistent with our speculation that PD-L1 negatively regulates the migration and invasion of trophoblasts by upregulating ARHGDIB.

PD-L1 is well known as a type 1 transmembrane glycoprotein and is mainly located within cellular membranes. Recent studies have indicated that PD-L1 can regulate gene expression, but very few studies have focused on activating transcription factors [21, 22]. Our RNA-sequencing data indicated that the transcription factor PU.1 (SPI1), an important member of the ETS family, was deeply involved in the regulation of differential gene expression. Most studies have mainly focused on the role of PU.1 in regulating cell differentiation, proliferation, and apoptosis [23–26]. Double luciferase reporter gene assays confirmed that ARHGDIB was a potential target gene of PU.1. Meanwhile, the migration and invasion of trophoblasts were significantly enhanced after PU.1 knockdown. The above findings strongly supported that the transcription factor PU.1 mediated the PD-L1-induced increase in ARHGDIB expression.

In summary, our data indicated that PD-L1 was abundantly expressed in trophoblasts of the human placenta throughout pregnancy. Notably, PD-L1 was not only associated with maternal-fetal immune tolerance but also deeply regulated trophoblasts cell migration and invasion, which was likely mediated through the transcription factor PU.1-induced ARHGDIB transcription. Ectopic expression of PD-L1 could directly induce trophoblasts dysfunction and lead to placenta-derived pregnancy disorders, such as PE. Therefore, this study provides a better understanding of the role of PD-L1 in placental growth and development during pregnancy and highlights that PD-L1 serves as a potential therapeutic target for placental disorders in the future.

## Supplementary information

Supplementary Table and Figure(PDF)

Supplementary Table 1.

Supplementary Table 2.

Supplementary Table 3.

Supplementary Figure 1

Supplementary Figure 2

Supplementary Figure 3

Supplementary Legends

Full and uncropped western blots

Full and uncropped western blots

Full and uncropped western blots

Full and uncropped western blots

Full and uncropped western blots

Full and uncropped western blots

Full and uncropped western blots

Full and uncropped western blots

Full and uncropped western blots

Full and uncropped western blots

Full and uncropped western blots

Full and uncropped western blots

Full and uncropped western blots

Full and uncropped western blots

Full and uncropped western blots

Full and uncropped western blots

Full and uncropped western blots

Full and uncropped western blots

Full and uncropped western blots

Full and uncropped western blots

Full and uncropped western blots

Full and uncropped western blots

Western Blots merge PDF

## Data Availability

All data included in this study are available upon request by contact with the corresponding author.
