# Supplementary material for: PD-L1 enhances migration and invasion of trophoblasts by upregulating ARHGDIB via transcription factor PU.1
Source: Cell Death Discov. 2022 Sep 22;8:395. doi: 10.1038/s41420-022-01171-6 (PMC9500068; doi:10.1038/s41420-022-01171-6)
Supplement: Supplementary file 1 — Supplementary Table and Figure(PDF) [file 41420_2022_1171_MOESM1_ESM.pdf]

S1.

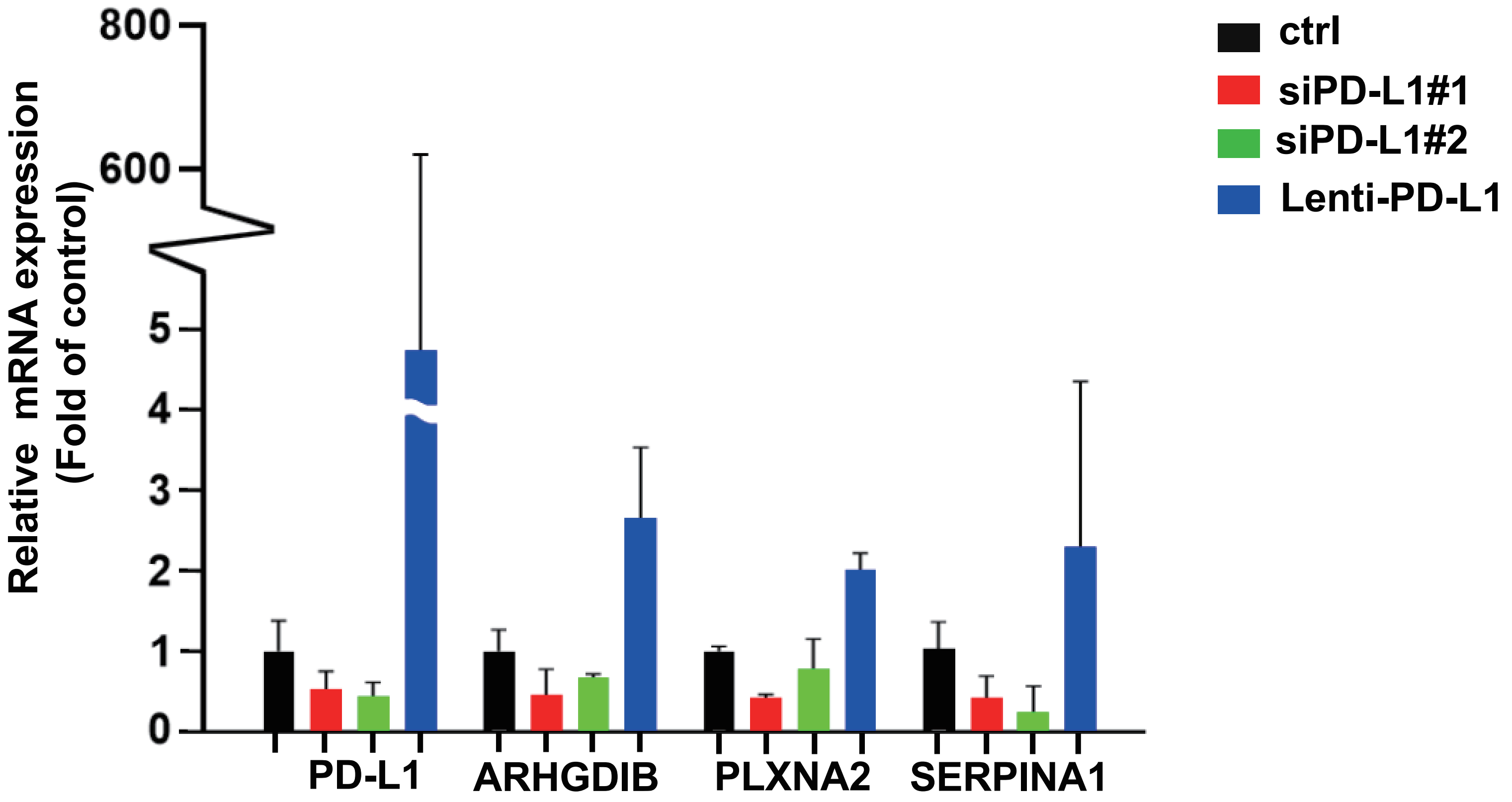

Fig. S1. The RT–qPCR results validated the significant expression level changes of the most significant genes (ARHGDIB, PLXNA2, and SERPINA1), which are the target genes of PD-L1 identified by RNA-seq.

S2.

A

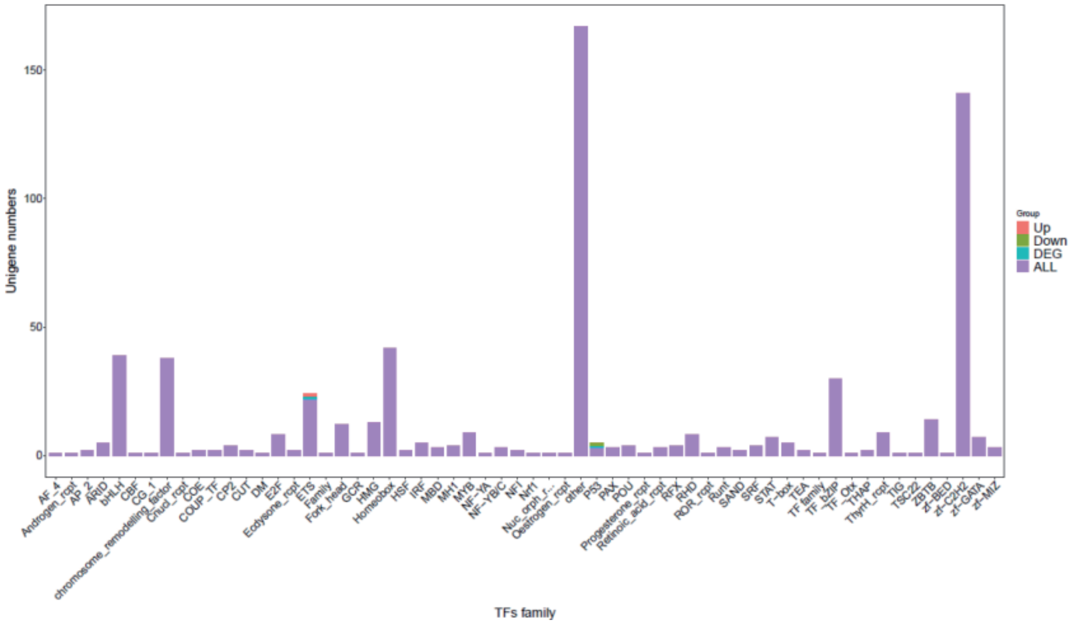

B

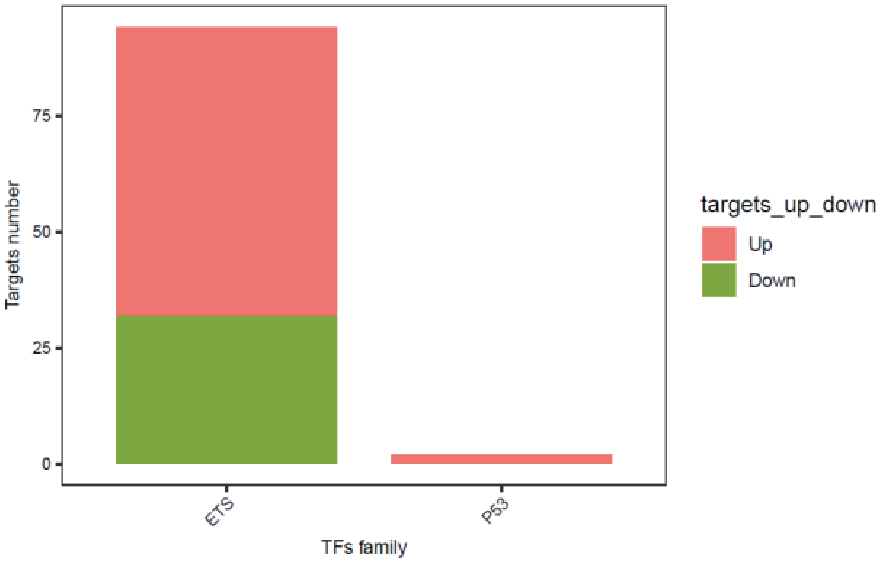

Fig. S2. The transcription factor PU.1 (SPI1) downstream of PD-L1 may affect the function of trophoblasts by regulating ARHGDIB. PU.1 (SPI1) belongs to the ETS family and is one of the proteins that are significantly upregulated in the PD-L1-overexpressing HTR-8/SVneo group.

S3.

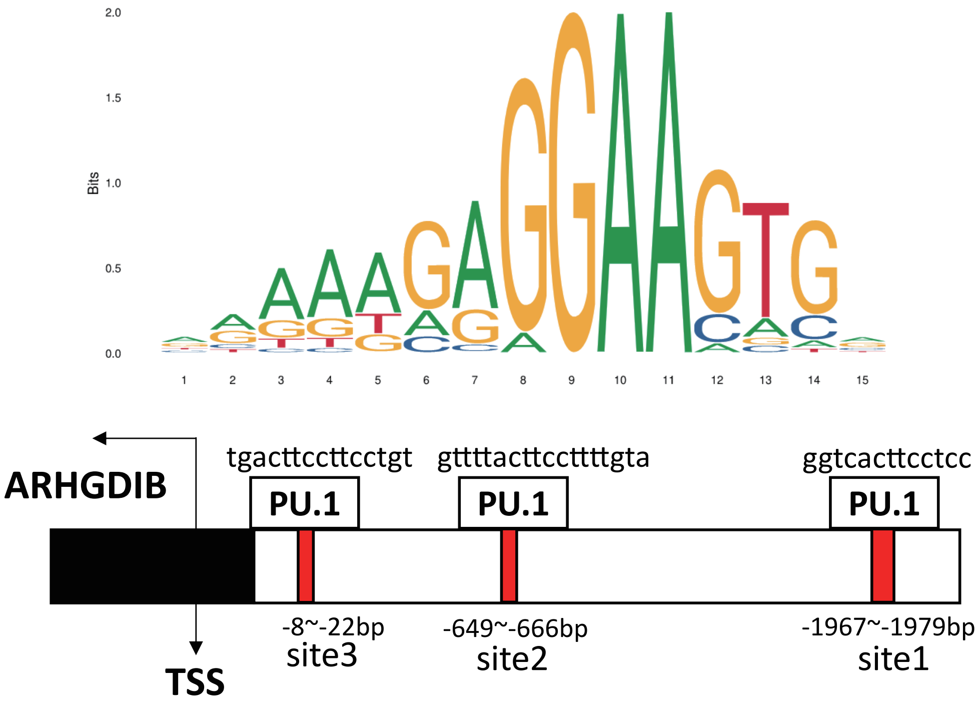

WT TCCCTCTTAACTGAGCGCAGTGGTCACTTCCTCCAAAAAGCCTTCCT.......ACTATGAAT
MT1 TCCCTCTTAACTGAGCGCAGTTTGACAGGAAGAAAAAAAGCCTTCCT.......ACTATGAAT

WT TAATGAAGTTTT ACTTCCTTTTGTAATTTCTTTCCTAGGTTTCATCAAAGACAGTACTTTC
MT2 TAATGAATGGGGCAGGAAGGGGTGCATTTCTTTCCTAGGTTTCATCAAAGACAGTACTTTC

WT GCTTGCTTG .............AACATTTTTTTTCTCATTGACTTCCTTCCTGTTCTAACT
MT3 GCTTGCTTG .............AACATTTTTTTTCTCATGTCAGGAAGGAAGTGTCTAACT

Fig. S3. The DNA motif of PU.1 and the predicted binding sites on the ARHGDIB promoter were obtained from JASPAR, hTFtarget, and PROMO. Three sites where the transcription factor PU.1 is most likely to bind to the ARHGDIB promoter region (2000 bp).

Supplementary Table 1. Western Blot Antibodies

Antibody	Information
anti-PD-L1	#13684T, 1:2000; CST
anti-ARHGDIB	16122-1-AP, 1:5000; Proteintech
anti-PU.1	ab76543, 1:5000; Abcam
anti-GAPDH	D190090-0100, 1:8000; BBI Life Sciences
anti-actin	1:8000; BBI Life Sciences
anti-rabbit-IgG	1:8000; BBI Life Sciences
anti-mouse-IgG	D110087-0025, 1:8000; BBI Life Sciences

Supplementary Table 2. Immunofluorescence Antibodies

Antibody	Information
anti-PD-L1	Ab213524,1:500; Abcam
Anti-HLA-G	Ab52454,1:200; Abcam
anti-rabbit-IgG	Ab150077, 1:500; Abcam
anti-mouse-IgG	Ab150116, 1:500; Abcam

Supplementary Table 3. PCR primer sequences

Gene	Forward primer (5’-3’)	Reverse primer (5’-3’)
PD-L1	TGGCATTTGCTGAACGCATTT	TGCAGCCAGGTCTAATTGTTTT
ARHGDIB	AGTTGAGAGACAGAGGCACC	TCAGGGACTTCTGTGGTGGA
SPI1	GCGACCATTACTGGGACTTCC	GGGTATCGAGGACGTGCAT
SERPINA1	GGAGGCTCAGATCCATGAAGG	GGTGTCCCCGAAGTTGACAG
PLXNA2	CTGAGAATCGTGACTGGACCT	GCTTATAGACCCGGTTGATGG
GAPDH	AGGGCTGCTTTTAACTCTGGT	CCCCACTTGATTTTGGAGGGA
ACTIN	AGCCTCGCCTTTGCCGAT	CTTCTGACCCATGCCCACC
