# Supplementary material for: PD-L1 enhances migration and invasion of trophoblasts by upregulating ARHGDIB via transcription factor PU.1
Source: Cell Death Discov. 2022 Sep 22;8:395. doi: 10.1038/s41420-022-01171-6 (PMC9500068; doi:10.1038/s41420-022-01171-6)
Supplement: Supplementary file 2 — Supplementary Table 1. [file 41420_2022_1171_MOESM2_ESM.docx]

Supplementary Table 1. Western Blot Antibodies

| **Antibody** | **Information** |
| --- | --- |
| anti-PD-L1 | #13684T, 1:2000; CST |
| anti-ARHGDIB | 16122-1-AP, 1:5000; Proteintech |
| anti-PU.1 | ab76543, 1:5000; Abcam |
| anti-GAPDH | D190090-0100, 1:8000; BBI Life Sciences |
| anti-actin | 1:8000; BBI Life Sciences |
| anti-rabbit-IgG | 1:8000; BBI Life Sciences |
| anti-mouse-IgG | D110087-0025, 1:8000; BBI Life Sciences |
