# Supplementary material for: PD-L1 enhances migration and invasion of trophoblasts by upregulating ARHGDIB via transcription factor PU.1
Source: Cell Death Discov. 2022 Sep 22;8:395. doi: 10.1038/s41420-022-01171-6 (PMC9500068; doi:10.1038/s41420-022-01171-6)
Supplement: Supplementary file 3 — Supplementary Table 2. [file 41420_2022_1171_MOESM3_ESM.docx]

Supplementary Table 2. Immunofluorescence Antibodies

| **Antibody** | **Information** |
| --- | --- |
| anti-PD-L1 | Ab213524,1:500; Abcam |
| Anti-HLA-G | Ab52454,1:200; Abcam |
| anti-rabbit-IgG | Ab150077, 1:500; Abcam |
| anti-mouse-IgG | Ab150116, 1:500; Abcam |
