# Supplementary material for: PD-L1 enhances migration and invasion of trophoblasts by upregulating ARHGDIB via transcription factor PU.1
Source: Cell Death Discov. 2022 Sep 22;8:395. doi: 10.1038/s41420-022-01171-6 (PMC9500068; doi:10.1038/s41420-022-01171-6)
Supplement: Supplementary file 4 — Supplementary Table 3. [file 41420_2022_1171_MOESM4_ESM.docx]

Supplementary Table 3. PCR primer sequences

| Gene | Forward primer (5’-3’) | Reverse primer (5’-3’) |
| --- | --- | --- |
| PD-L1 | TGGCATTTGCTGAACGCATTT | TGCAGCCAGGTCTAATTGTTTT |
| ARHGDIB | AGTTGAGAGACAGAGGCACC | TCAGGGACTTCTGTGGTGGA |
| SPI1 | GCGACCATTACTGGGACTTCC | GGGTATCGAGGACGTGCAT |
| SERPINA1 | GGAGGCTCAGATCCATGAAGG | GGTGTCCCCGAAGTTGACAG |
| PLXNA2 | CTGAGAATCGTGACTGGACCT | GCTTATAGACCCGGTTGATGG |
| GAPDH | AGGGCTGCTTTTAACTCTGGT | CCCCACTTGATTTTGGAGGGA |
| ACTIN | AGCCTCGCCTTTGCCGAT | CTTCTGACCCATGCCCACC |
