# Supplementary figures and images for: PD-L1 enhances migration and invasion of trophoblasts by upregulating ARHGDIB via transcription factor PU.1
Source: Cell Death Discov. 2022 Sep 22;8:395. doi: 10.1038/s41420-022-01171-6 (PMC9500068; doi:10.1038/s41420-022-01171-6)

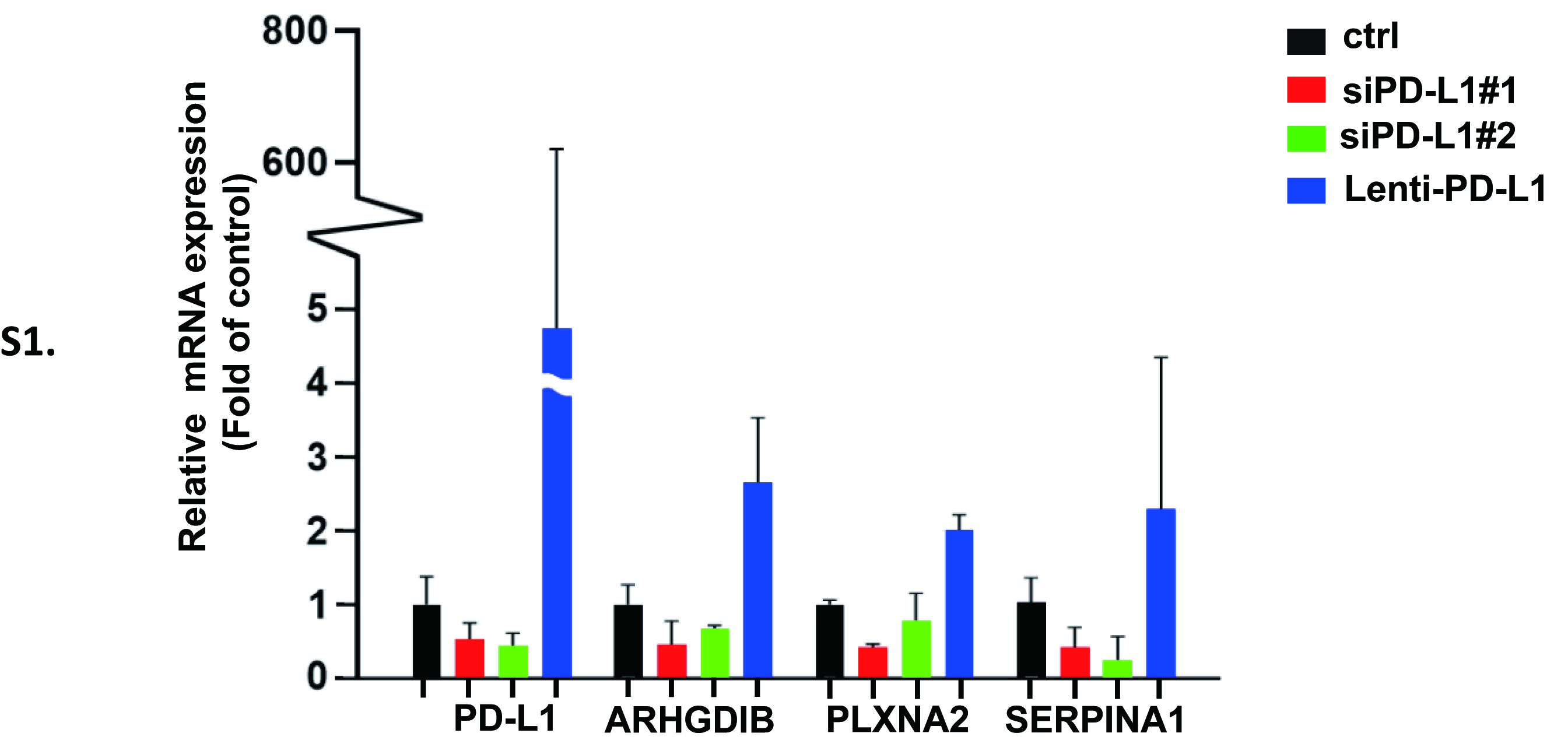

Supplement: Supplementary file 5 — Supplementary Figure 1 [file 41420_2022_1171_MOESM5_ESM.tif]

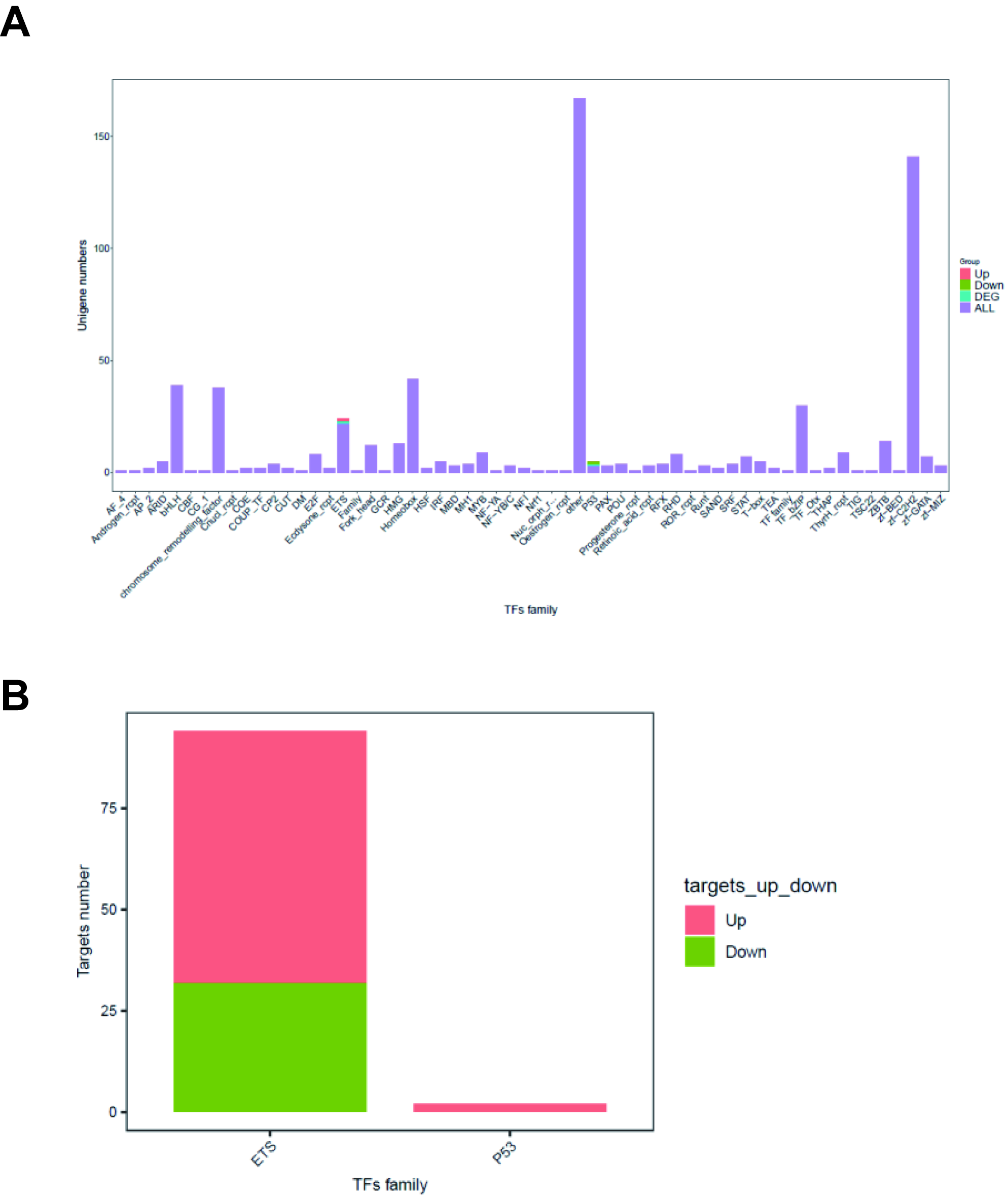

Supplement: Supplementary file 6 — Supplementary Figure 2 [file 41420_2022_1171_MOESM6_ESM.tif]

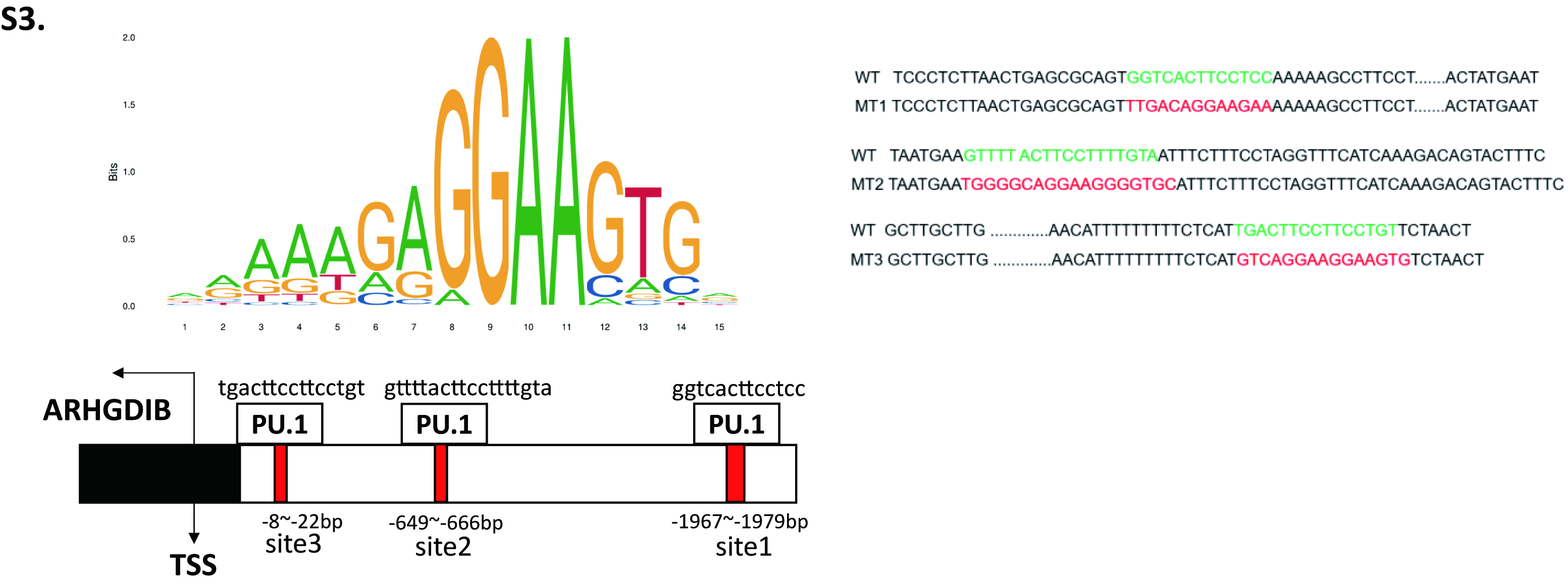

Supplement: Supplementary file 7 — Supplementary Figure 3 [file 41420_2022_1171_MOESM7_ESM.tif]

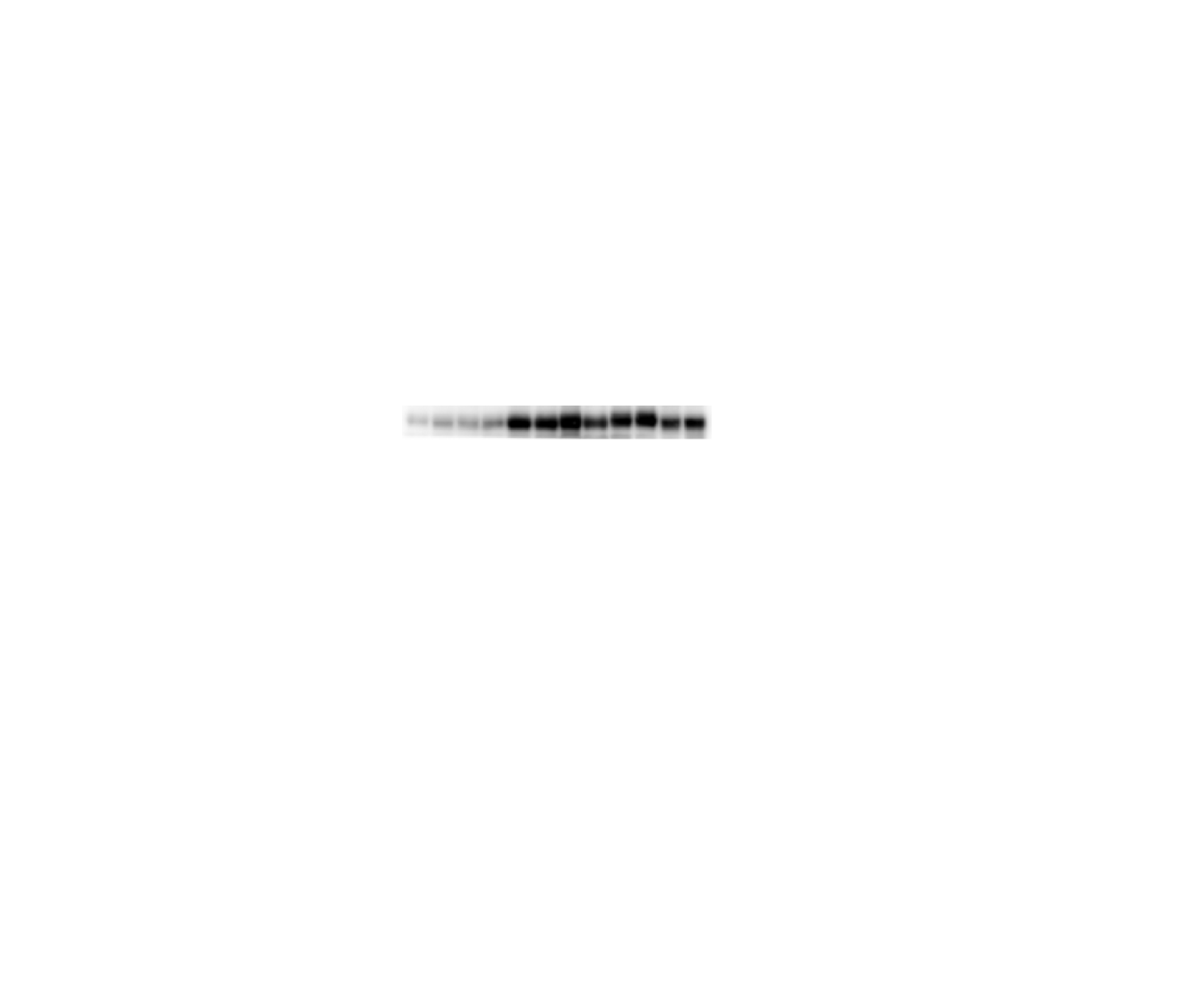

Supplement: Supplementary file 9 — Full and uncropped western blots [file 41420_2022_1171_MOESM9_ESM.tif]

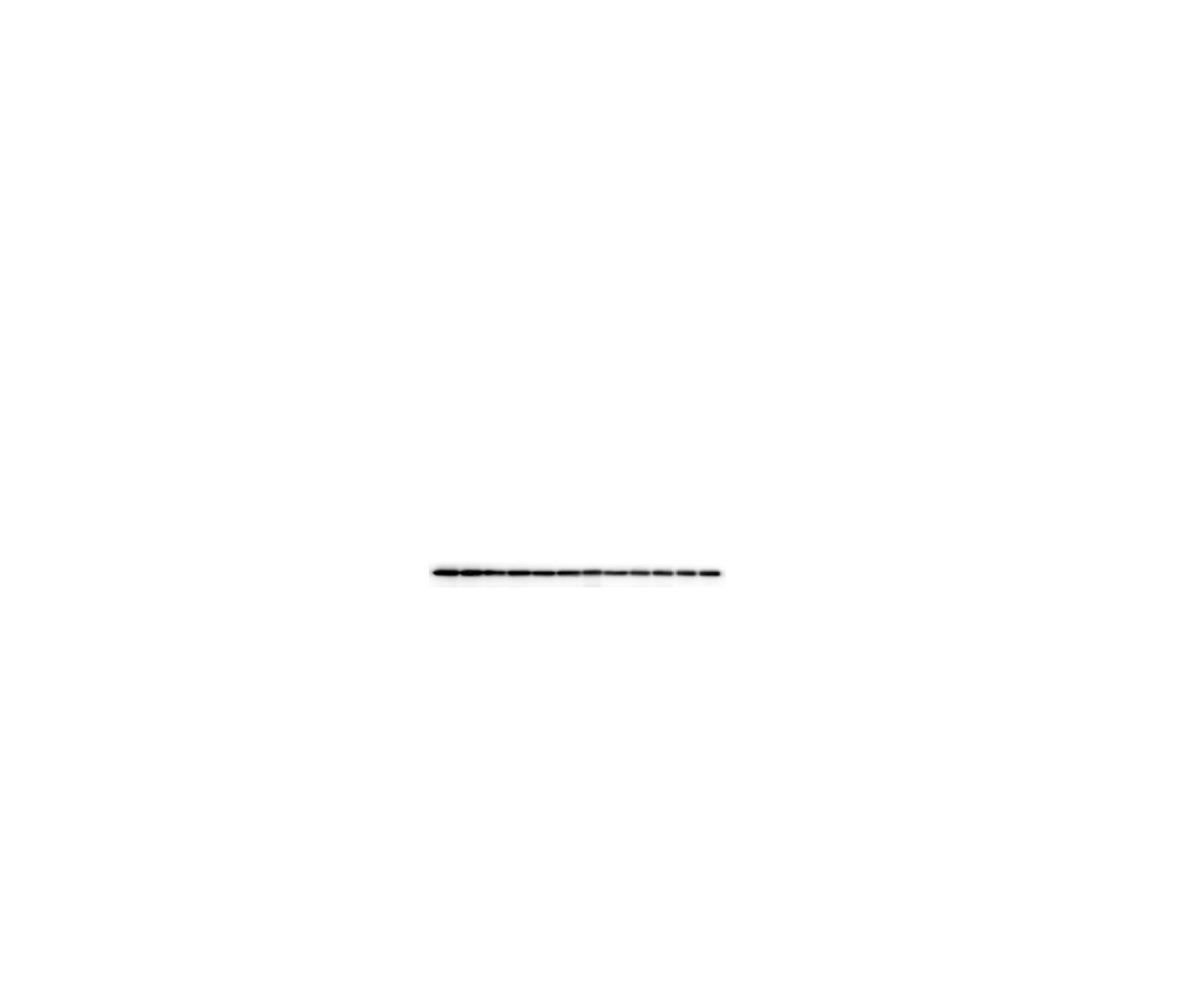

Supplement: Supplementary file 10 — Full and uncropped western blots [file 41420_2022_1171_MOESM10_ESM.tif]

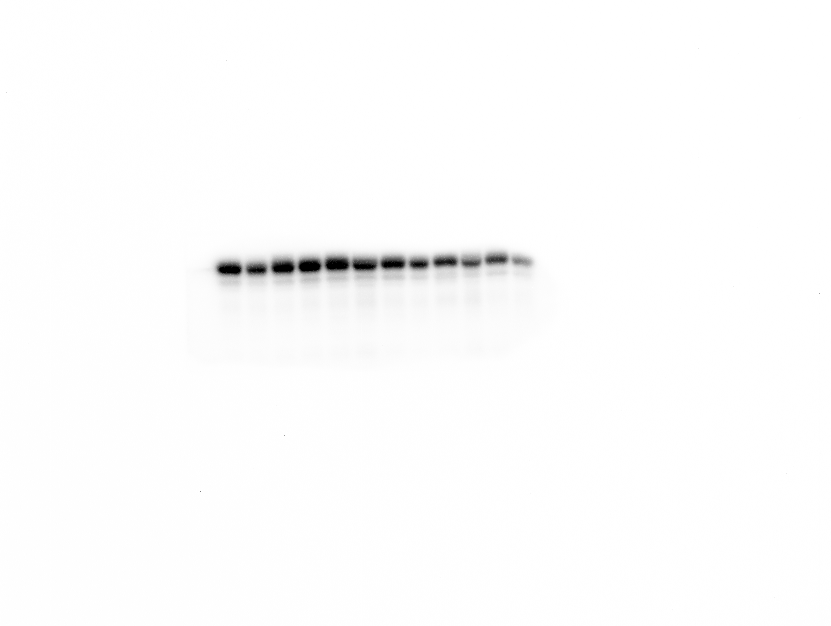

Supplement: Supplementary file 11 — Full and uncropped western blots [file 41420_2022_1171_MOESM11_ESM.png]

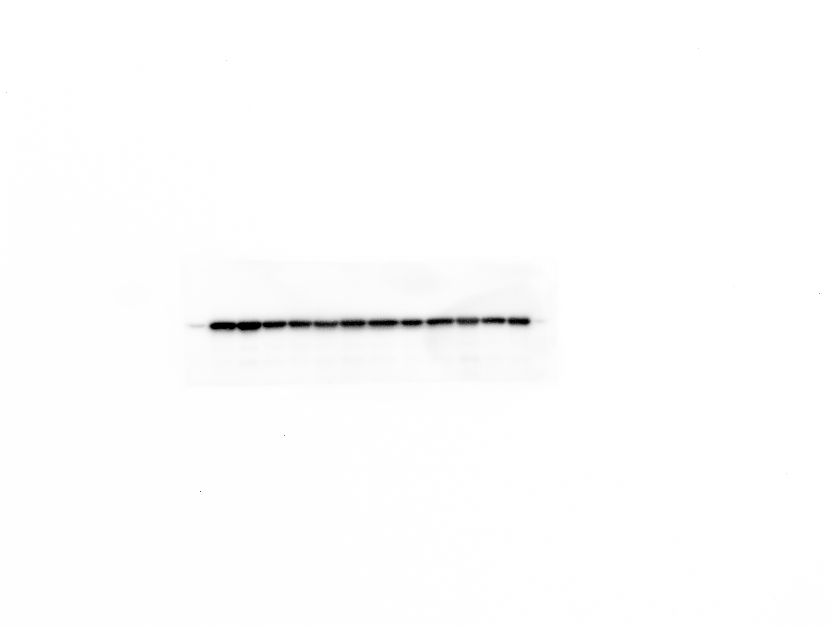

Supplement: Supplementary file 12 — Full and uncropped western blots [file 41420_2022_1171_MOESM12_ESM.png]

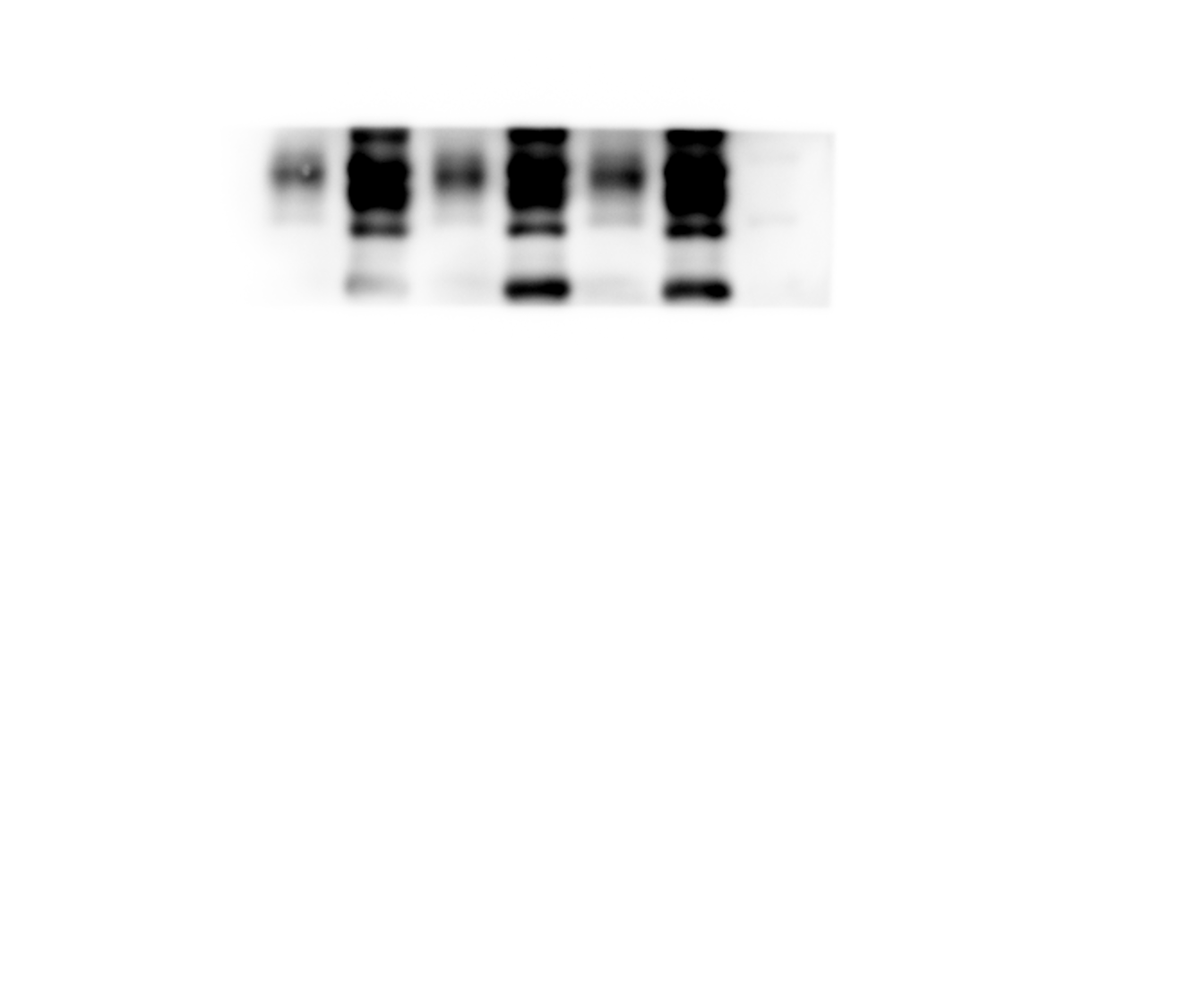

Supplement: Supplementary file 13 — Full and uncropped western blots [file 41420_2022_1171_MOESM13_ESM.tif]

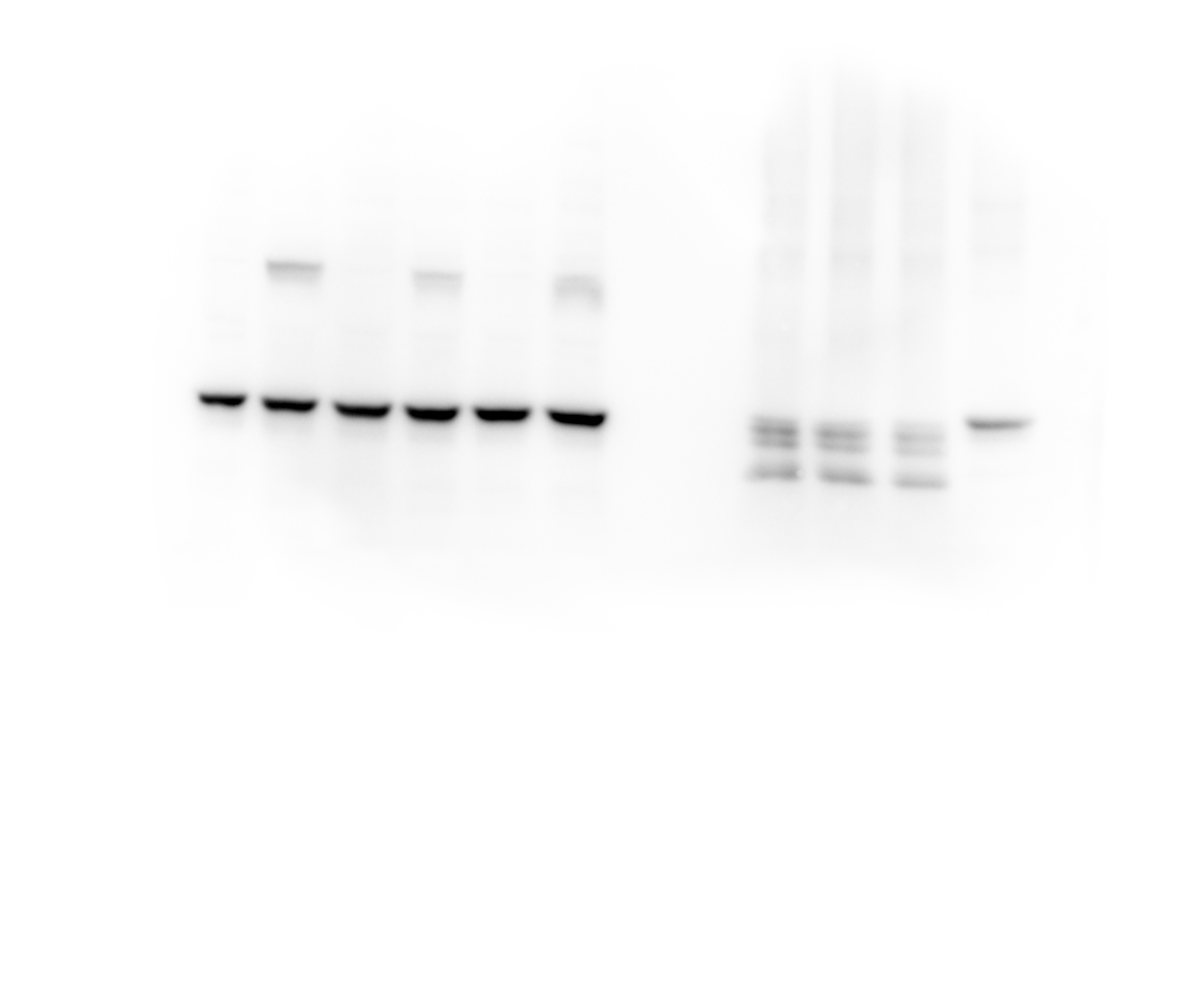

Supplement: Supplementary file 14 — Full and uncropped western blots [file 41420_2022_1171_MOESM14_ESM.tif]

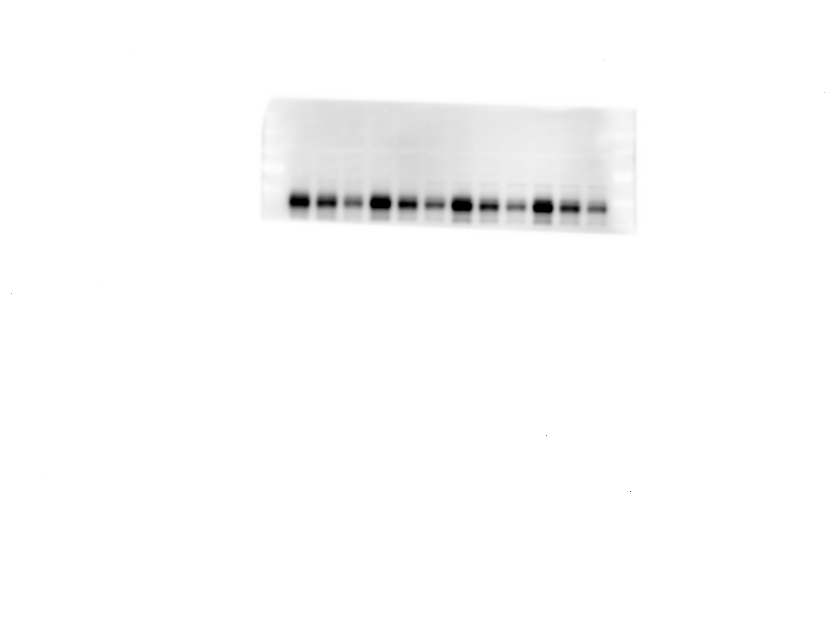

Supplement: Supplementary file 15 — Full and uncropped western blots [file 41420_2022_1171_MOESM15_ESM.png]

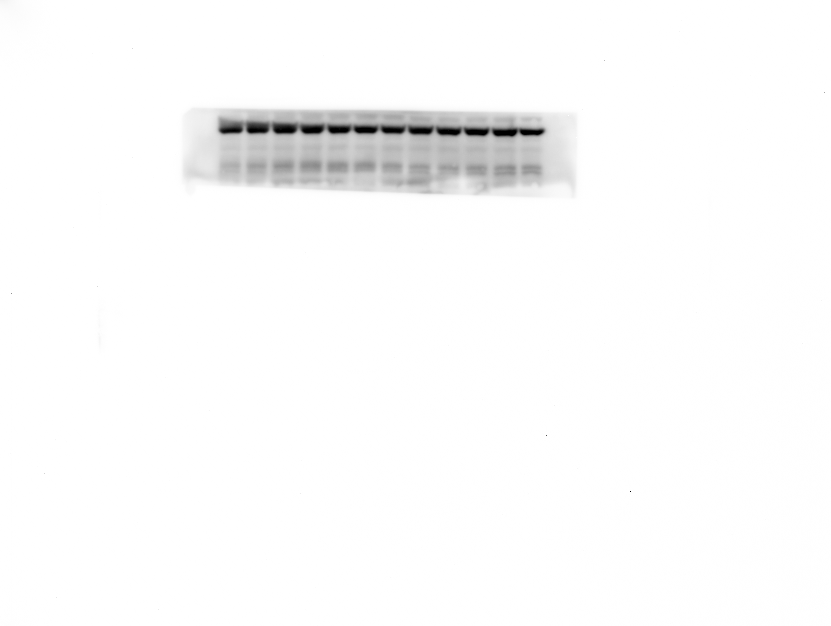

Supplement: Supplementary file 16 — Full and uncropped western blots [file 41420_2022_1171_MOESM16_ESM.png]

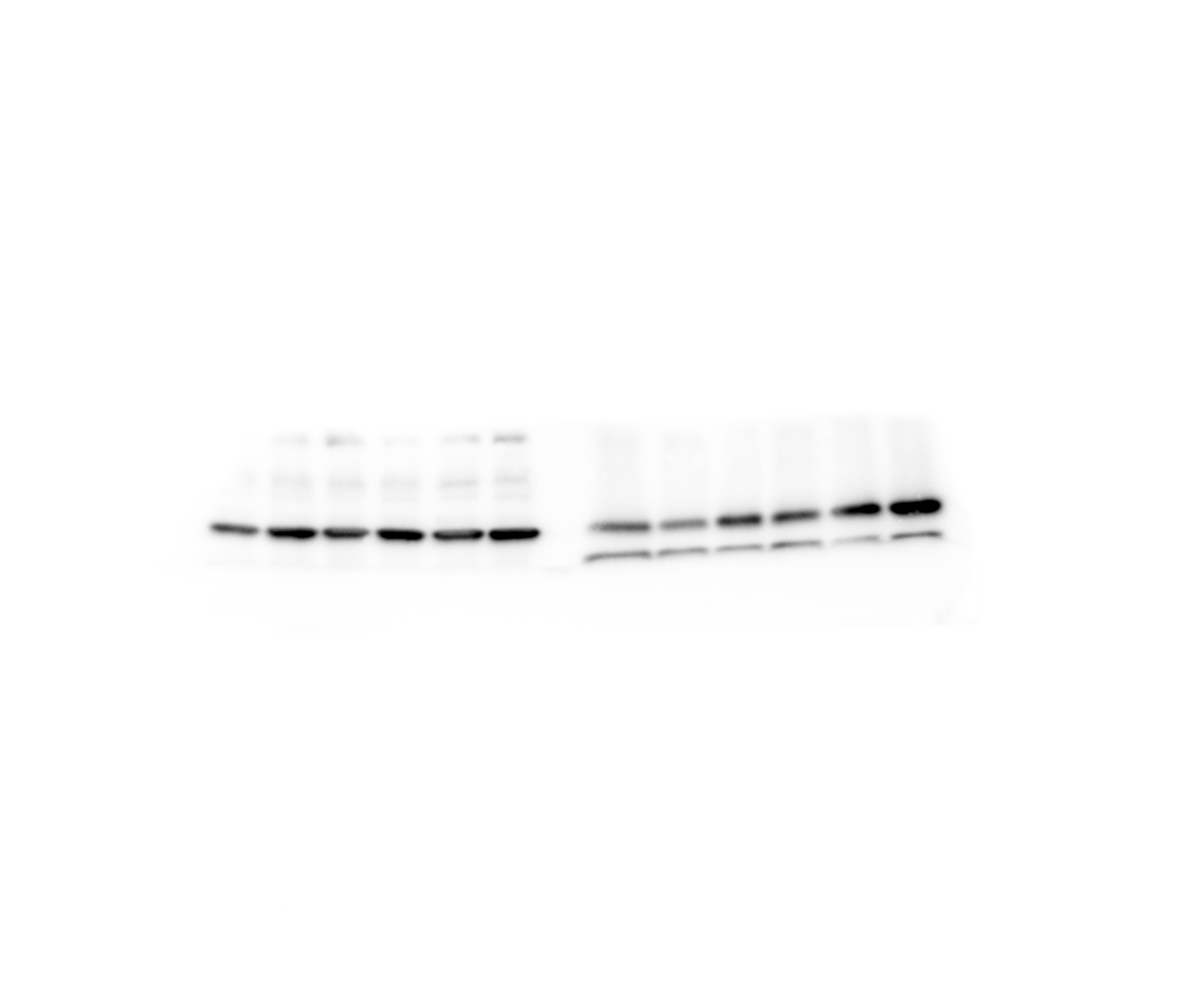

Supplement: Supplementary file 17 — Full and uncropped western blots [file 41420_2022_1171_MOESM17_ESM.tif]

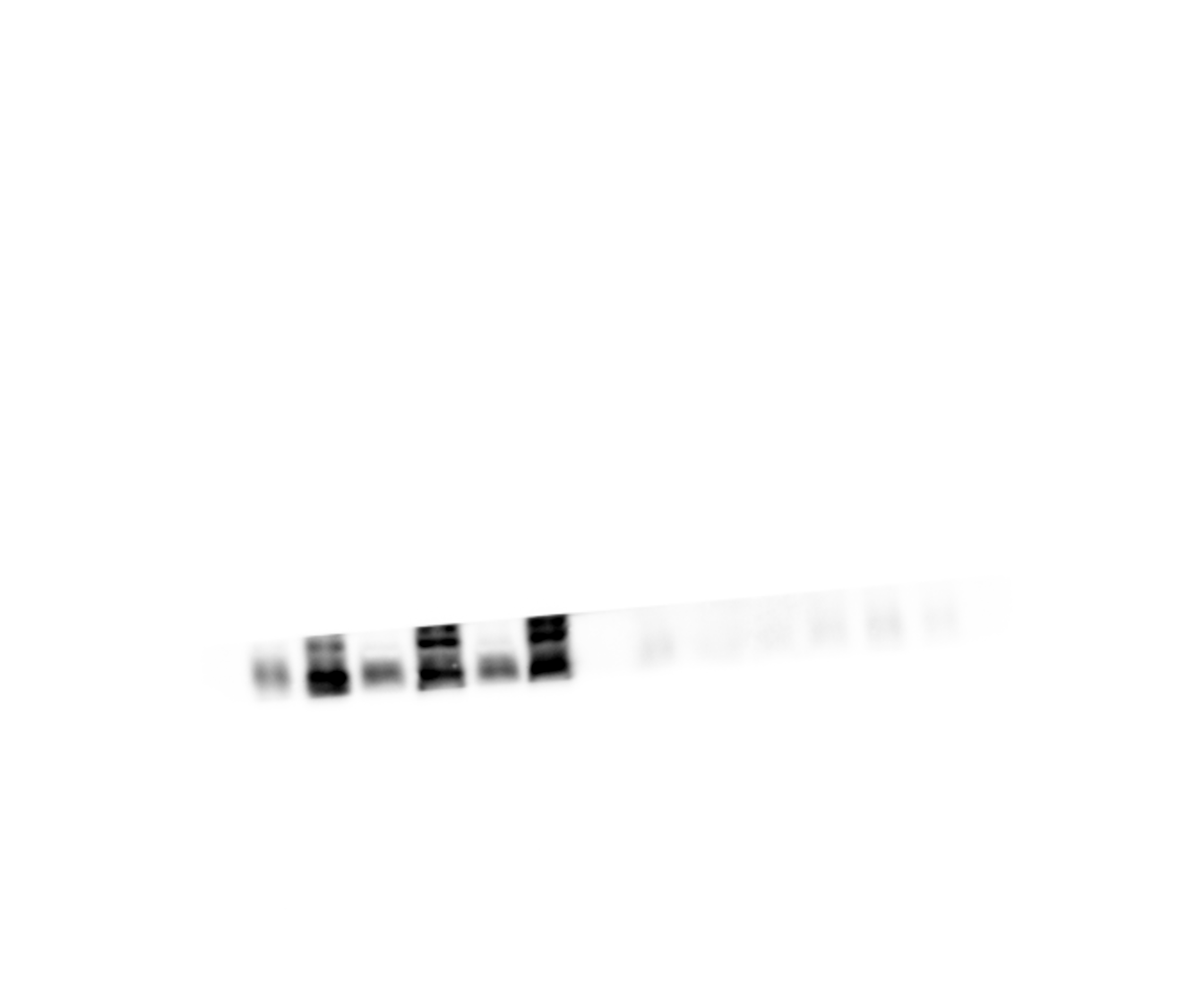

Supplement: Supplementary file 18 — Full and uncropped western blots [file 41420_2022_1171_MOESM18_ESM.tif]

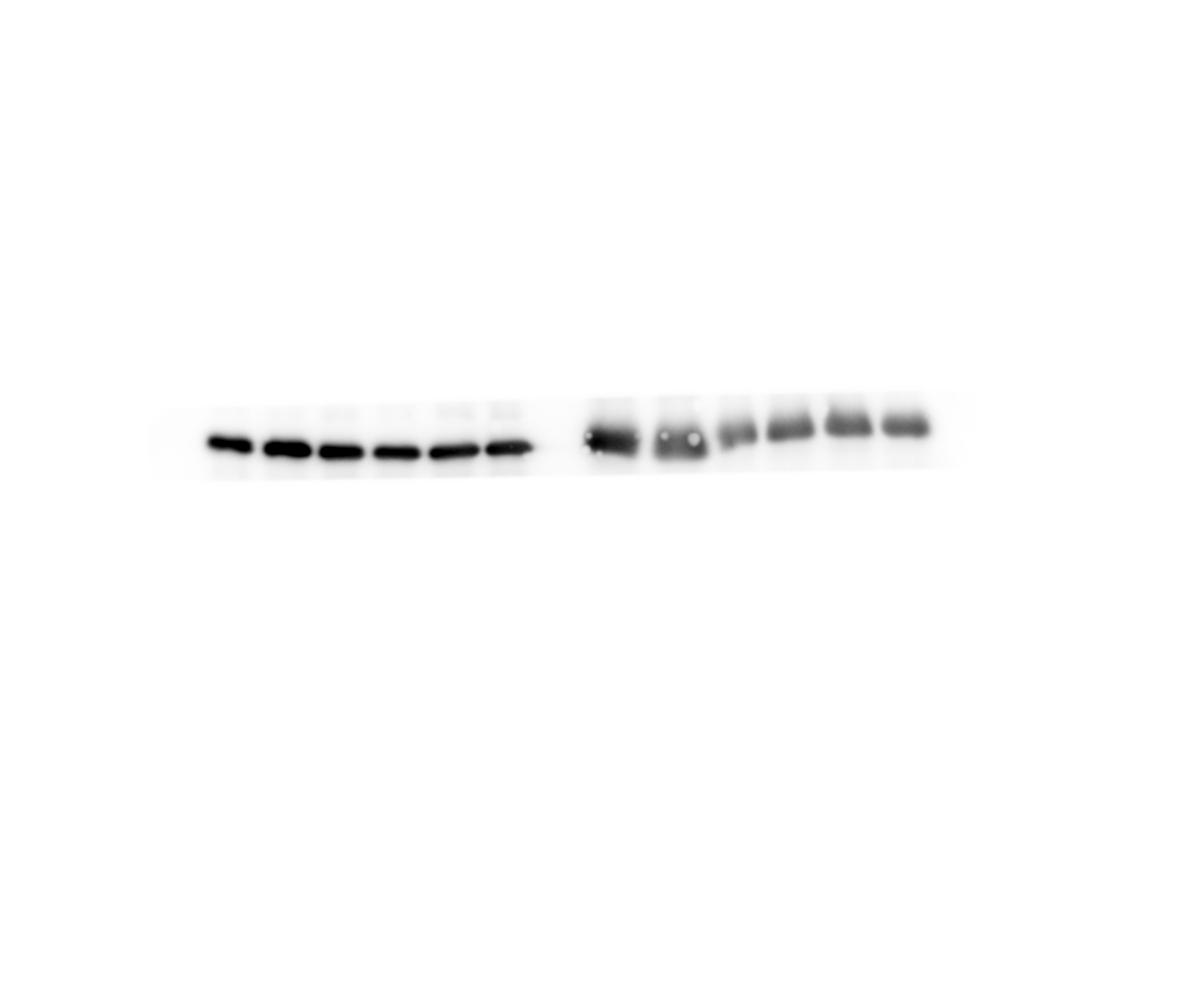

Supplement: Supplementary file 19 — Full and uncropped western blots [file 41420_2022_1171_MOESM19_ESM.tif]

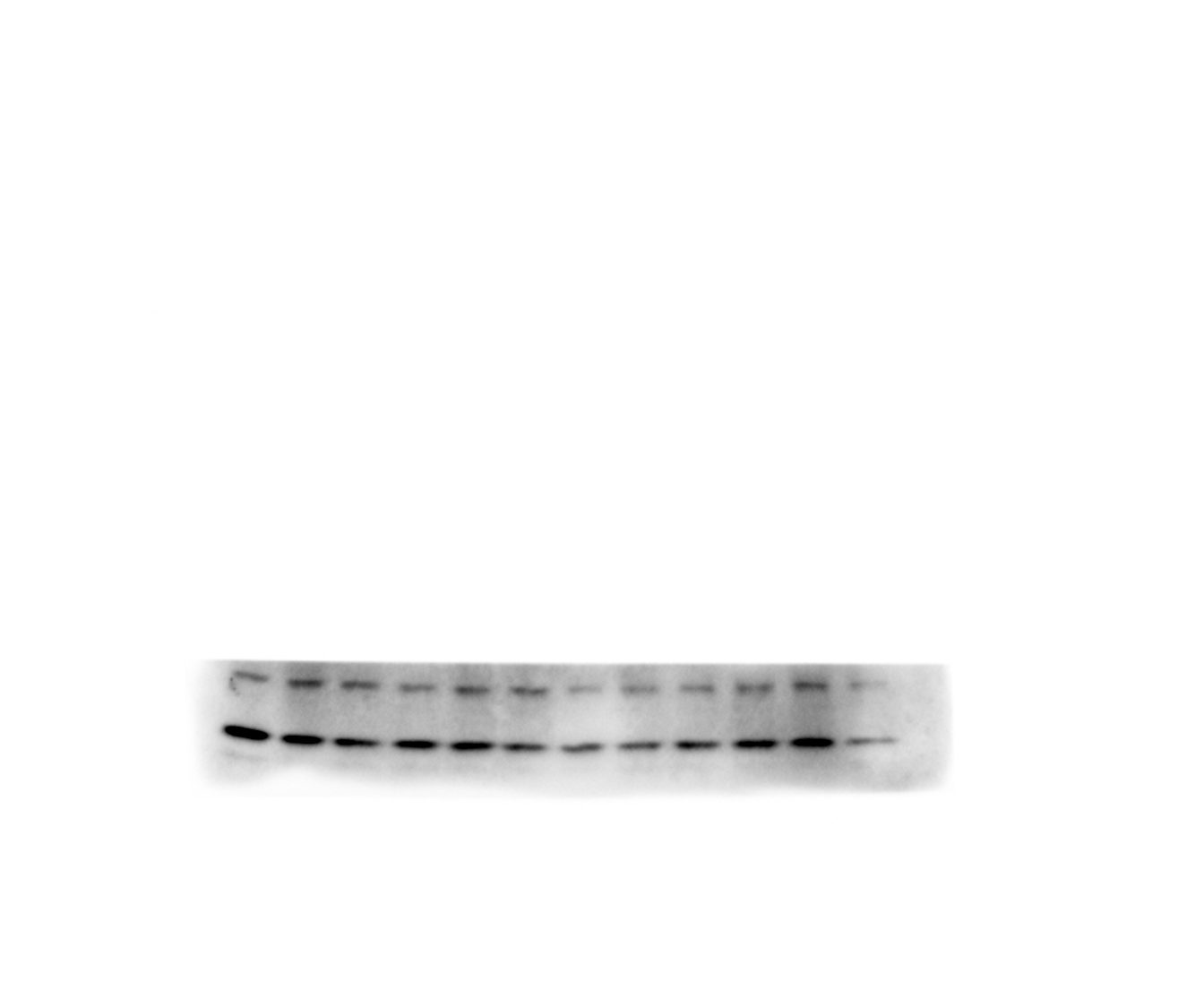

Supplement: Supplementary file 20 — Full and uncropped western blots [file 41420_2022_1171_MOESM20_ESM.tif]

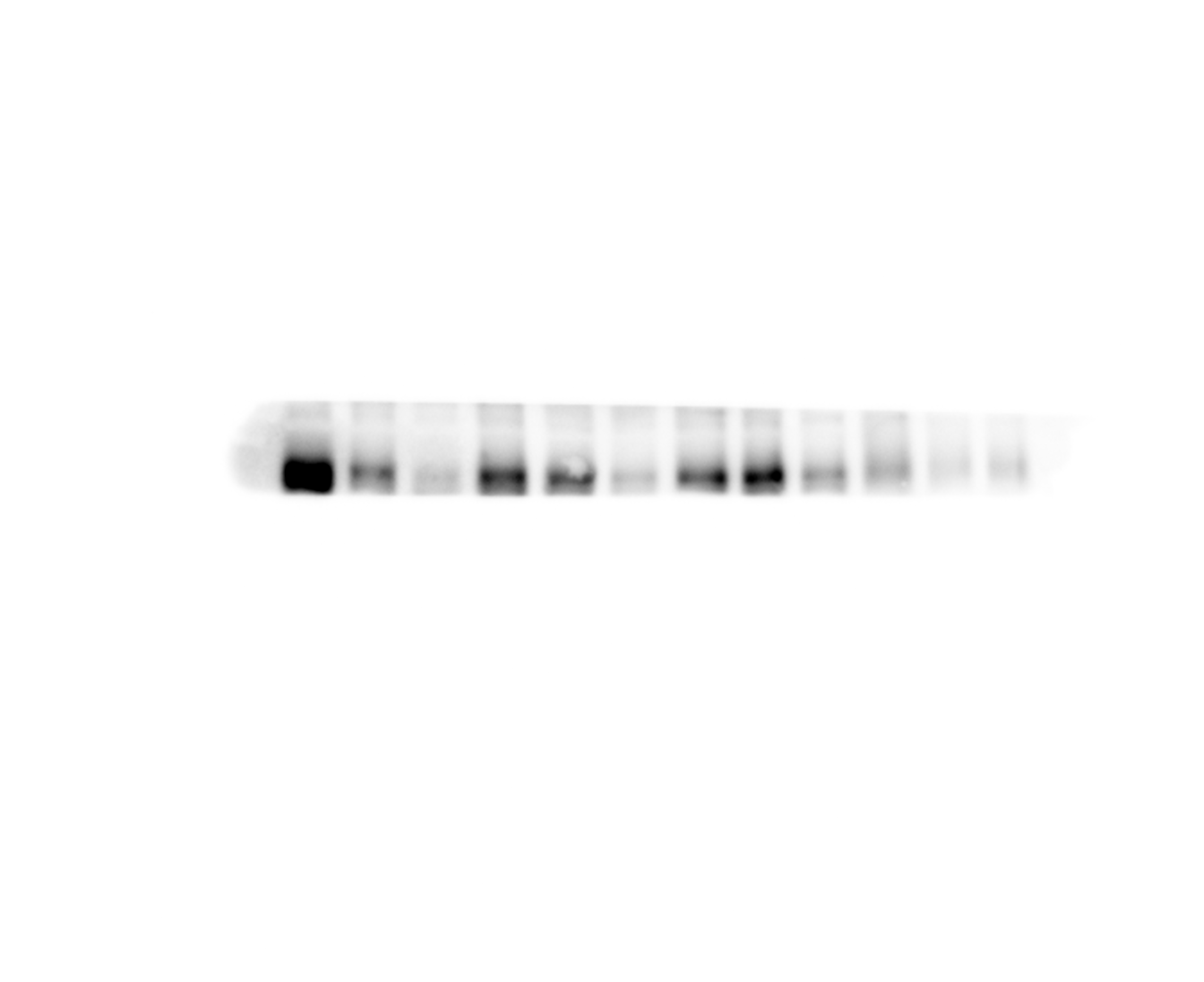

Supplement: Supplementary file 21 — Full and uncropped western blots [file 41420_2022_1171_MOESM21_ESM.tif]

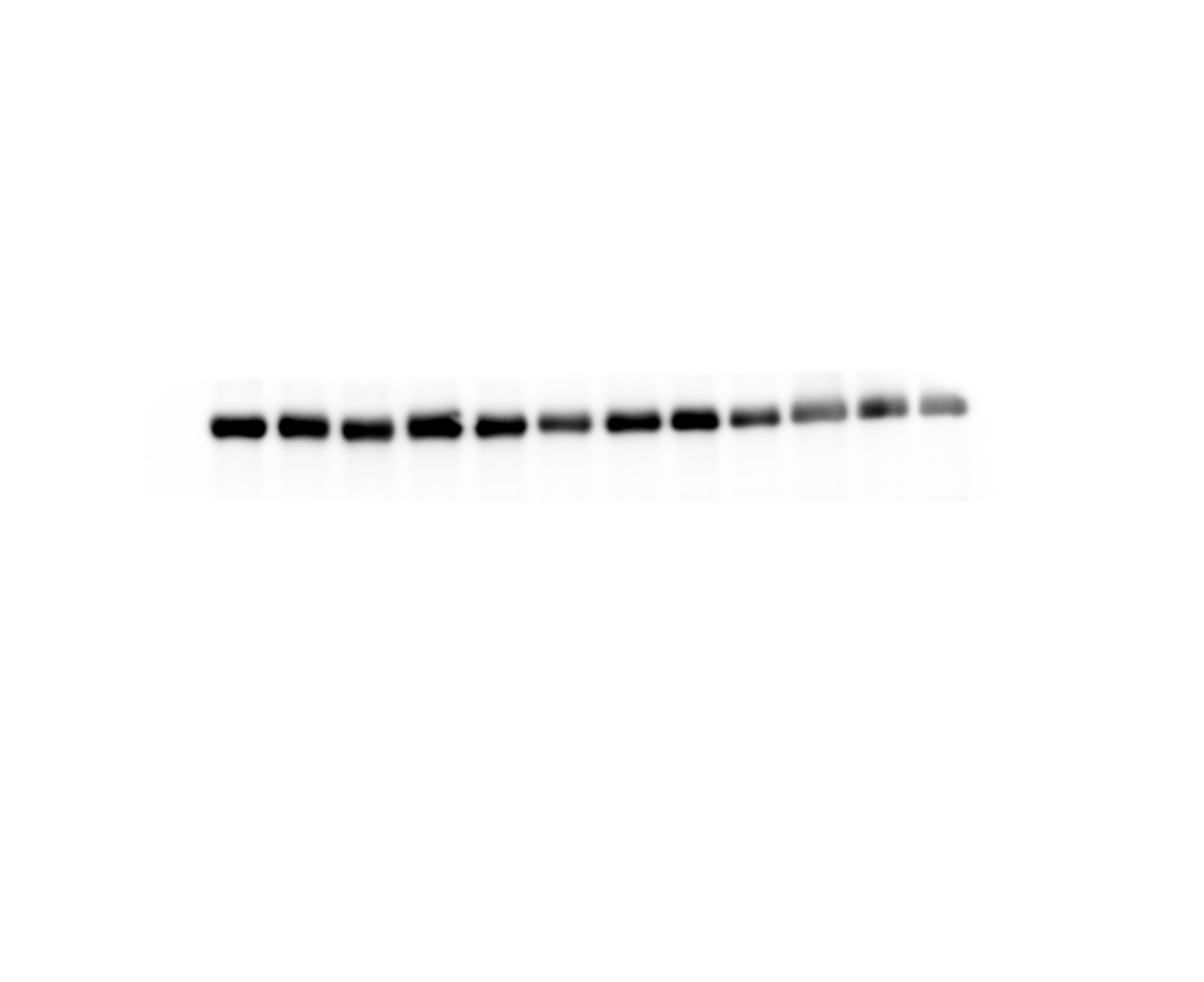

Supplement: Supplementary file 22 — Full and uncropped western blots [file 41420_2022_1171_MOESM22_ESM.tif]

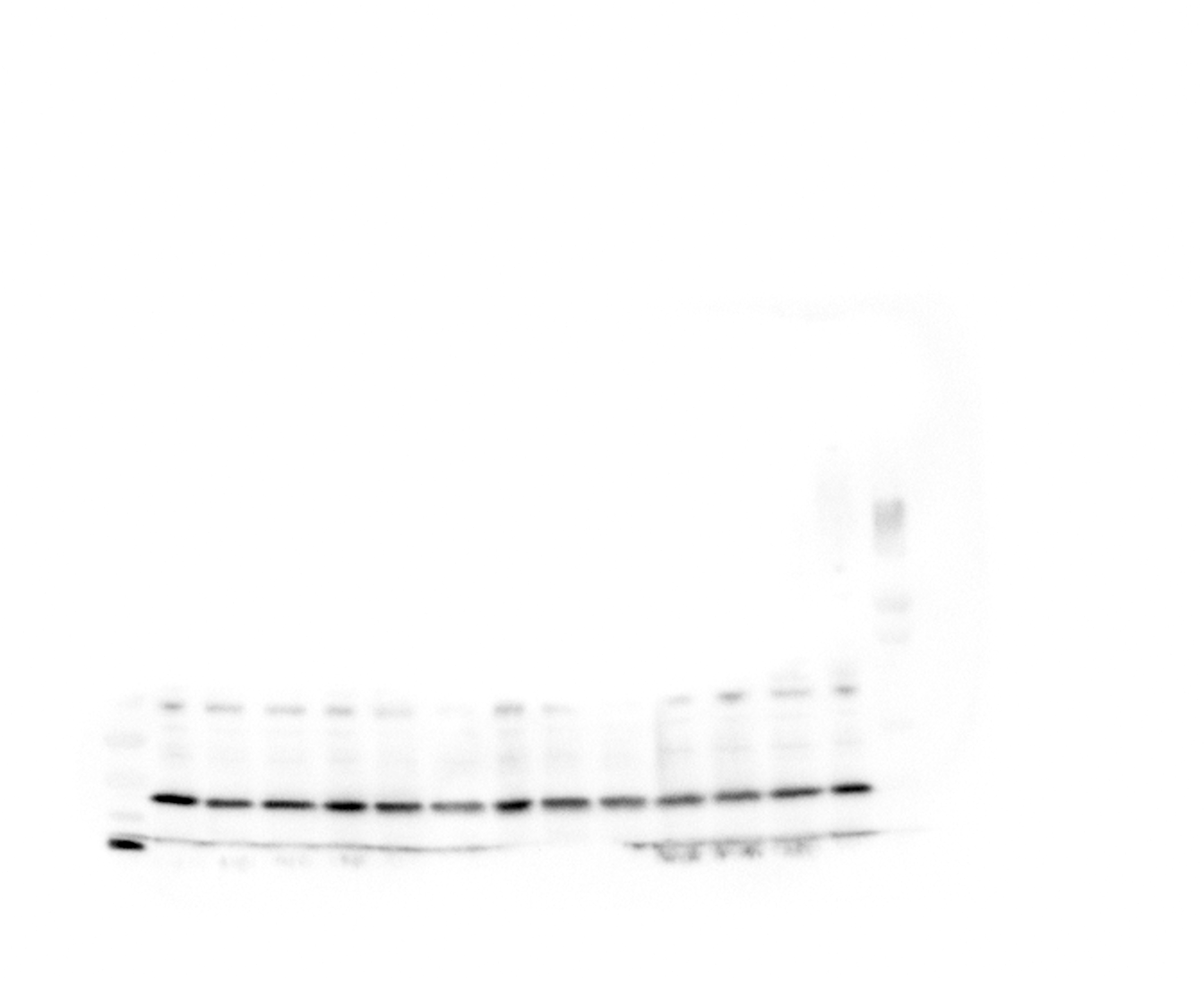

Supplement: Supplementary file 23 — Full and uncropped western blots [file 41420_2022_1171_MOESM23_ESM.tif]

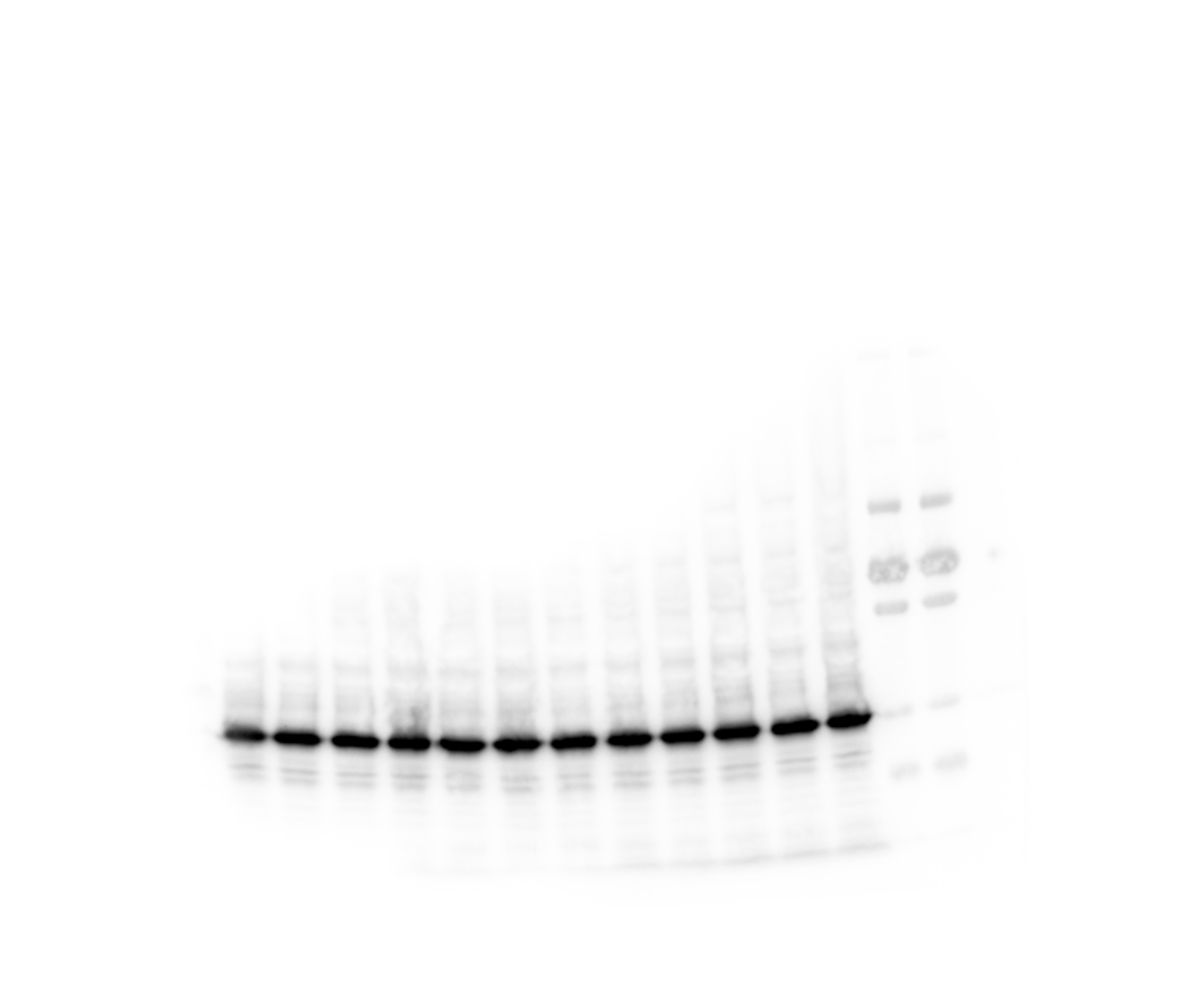

Supplement: Supplementary file 24 — Full and uncropped western blots [file 41420_2022_1171_MOESM24_ESM.tif]

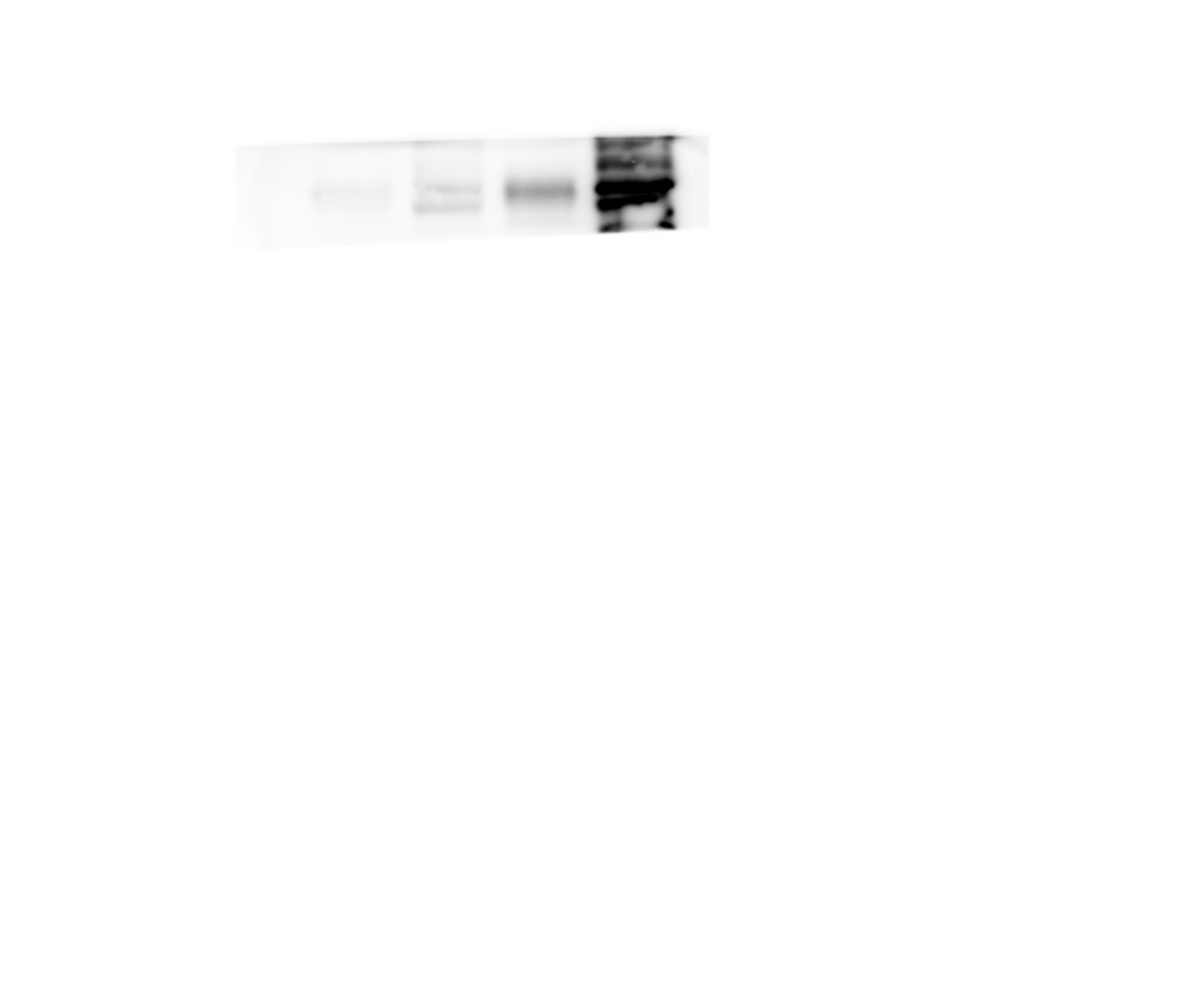

Supplement: Supplementary file 25 — Full and uncropped western blots [file 41420_2022_1171_MOESM25_ESM.tif]

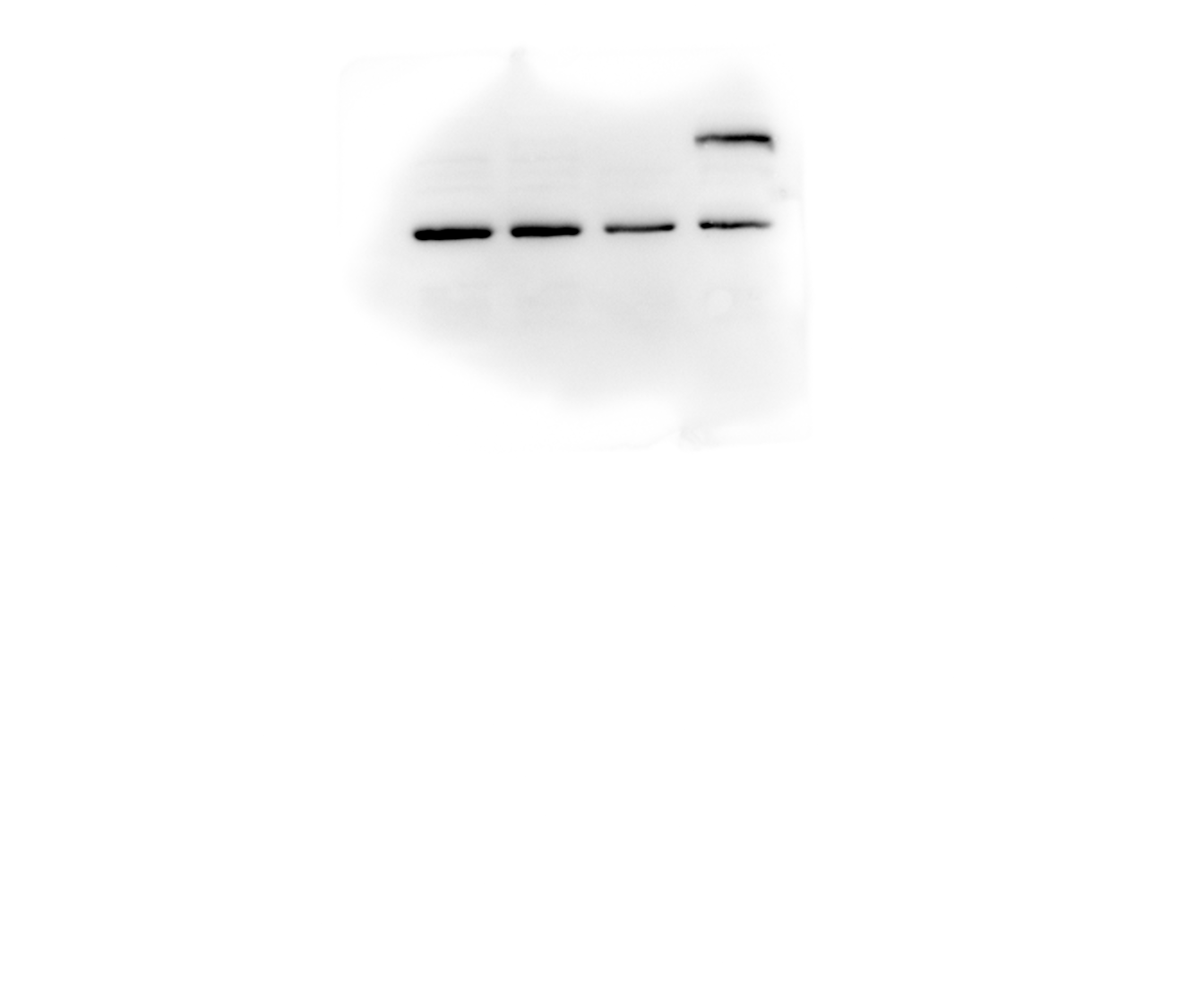

Supplement: Supplementary file 26 — Full and uncropped western blots [file 41420_2022_1171_MOESM26_ESM.tif]

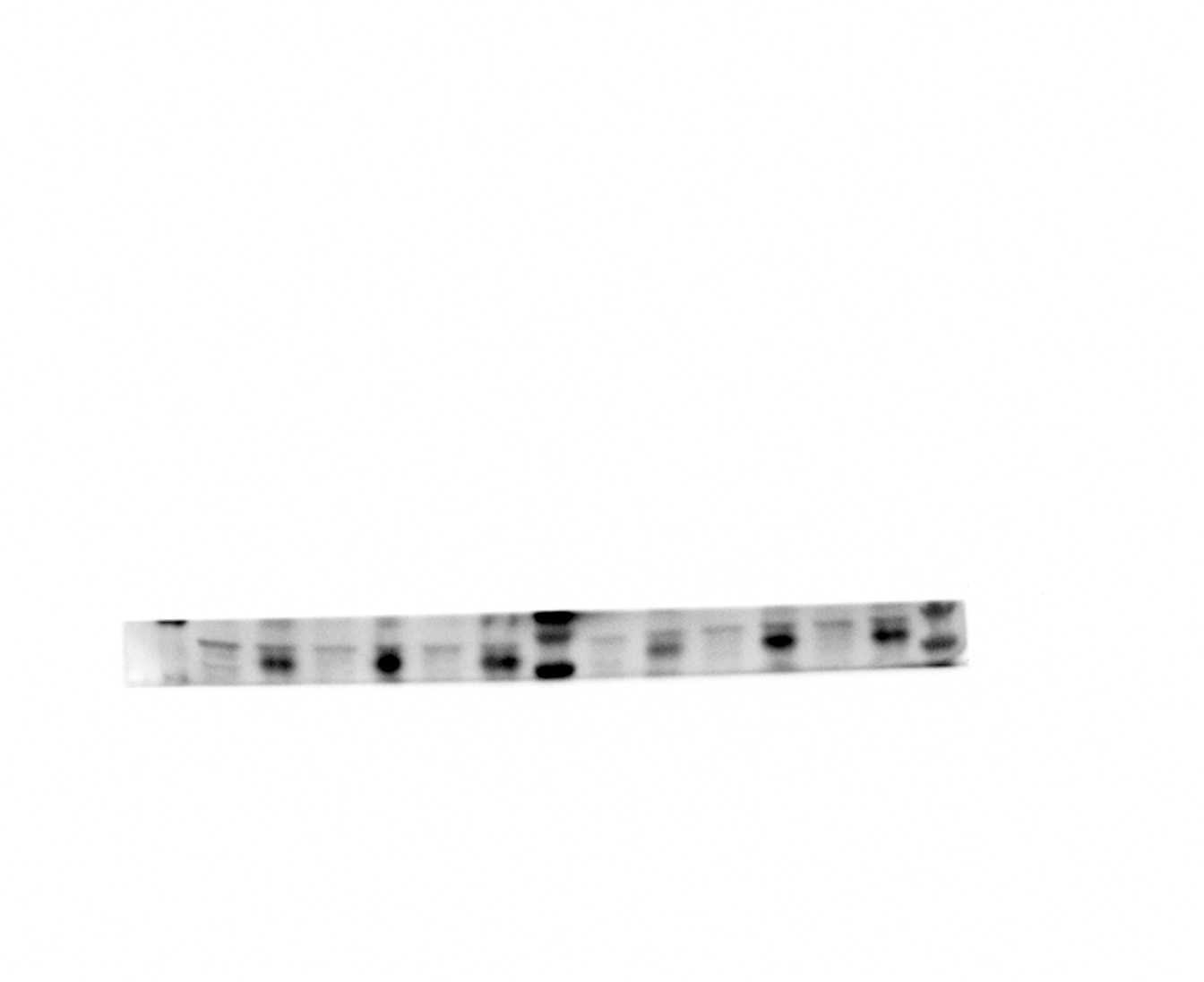

Supplement: Supplementary file 27 — Full and uncropped western blots [file 41420_2022_1171_MOESM27_ESM.tif]

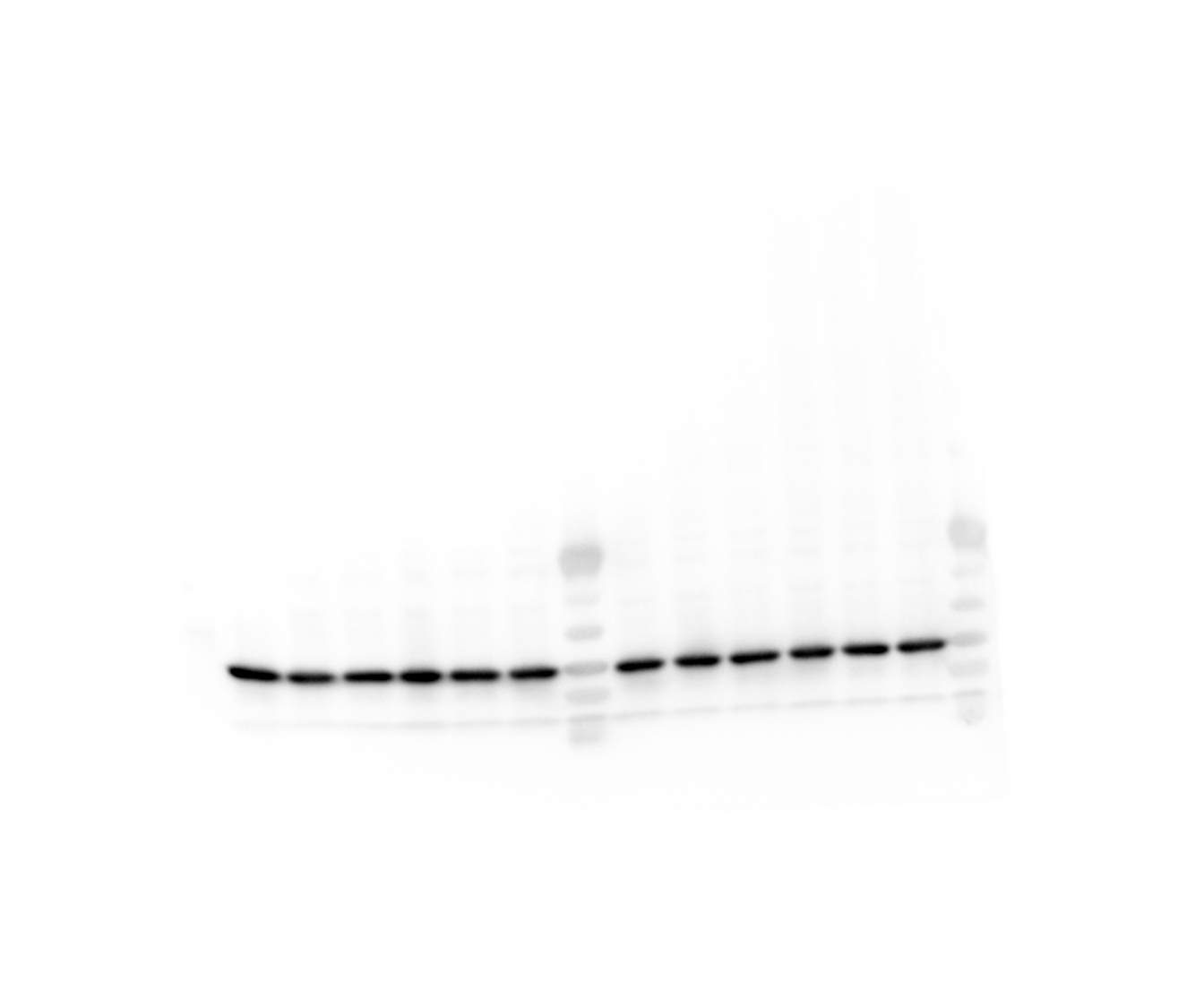

Supplement: Supplementary file 28 — Full and uncropped western blots [file 41420_2022_1171_MOESM28_ESM.tif]

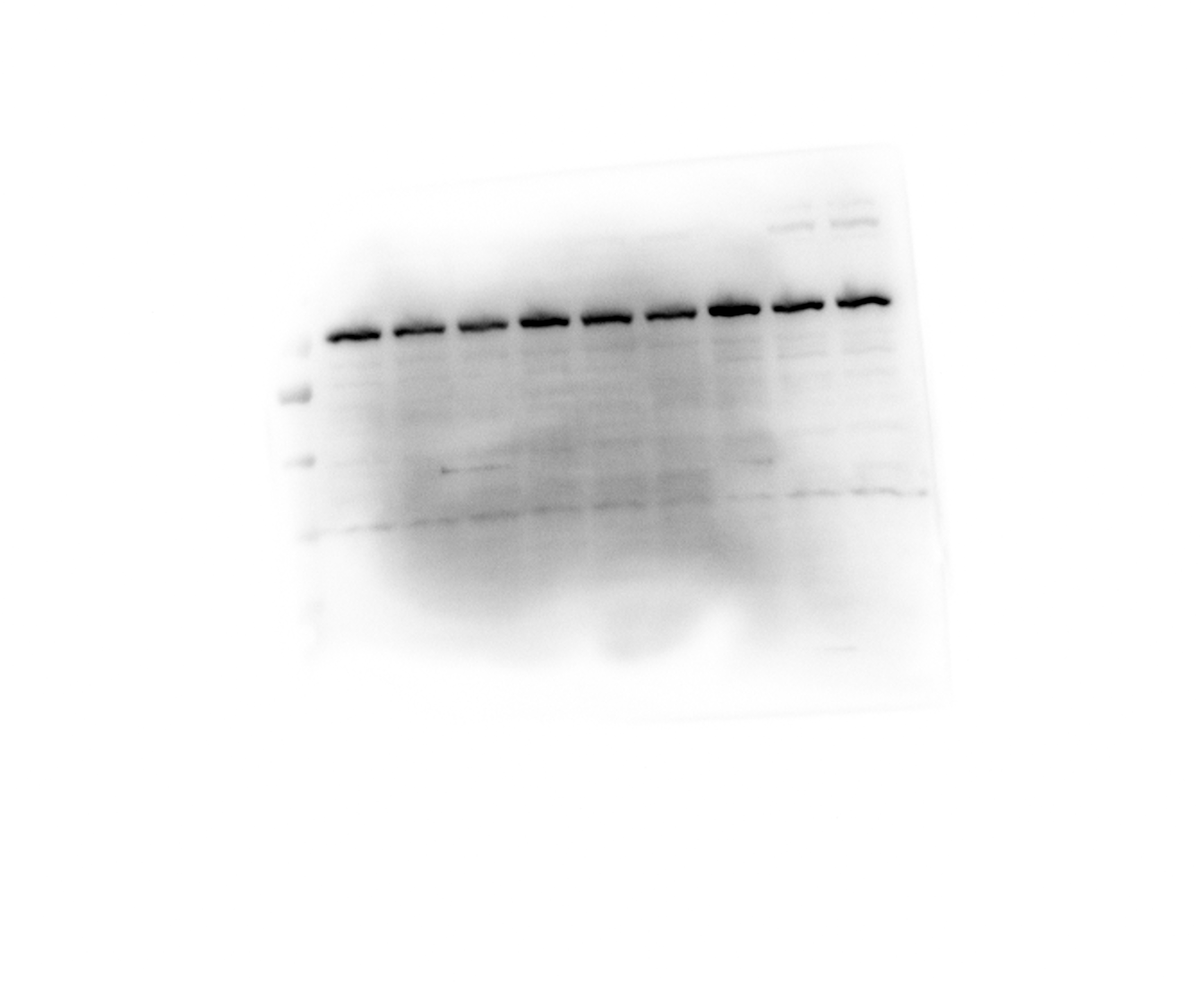

Supplement: Supplementary file 29 — Full and uncropped western blots [file 41420_2022_1171_MOESM29_ESM.tif]

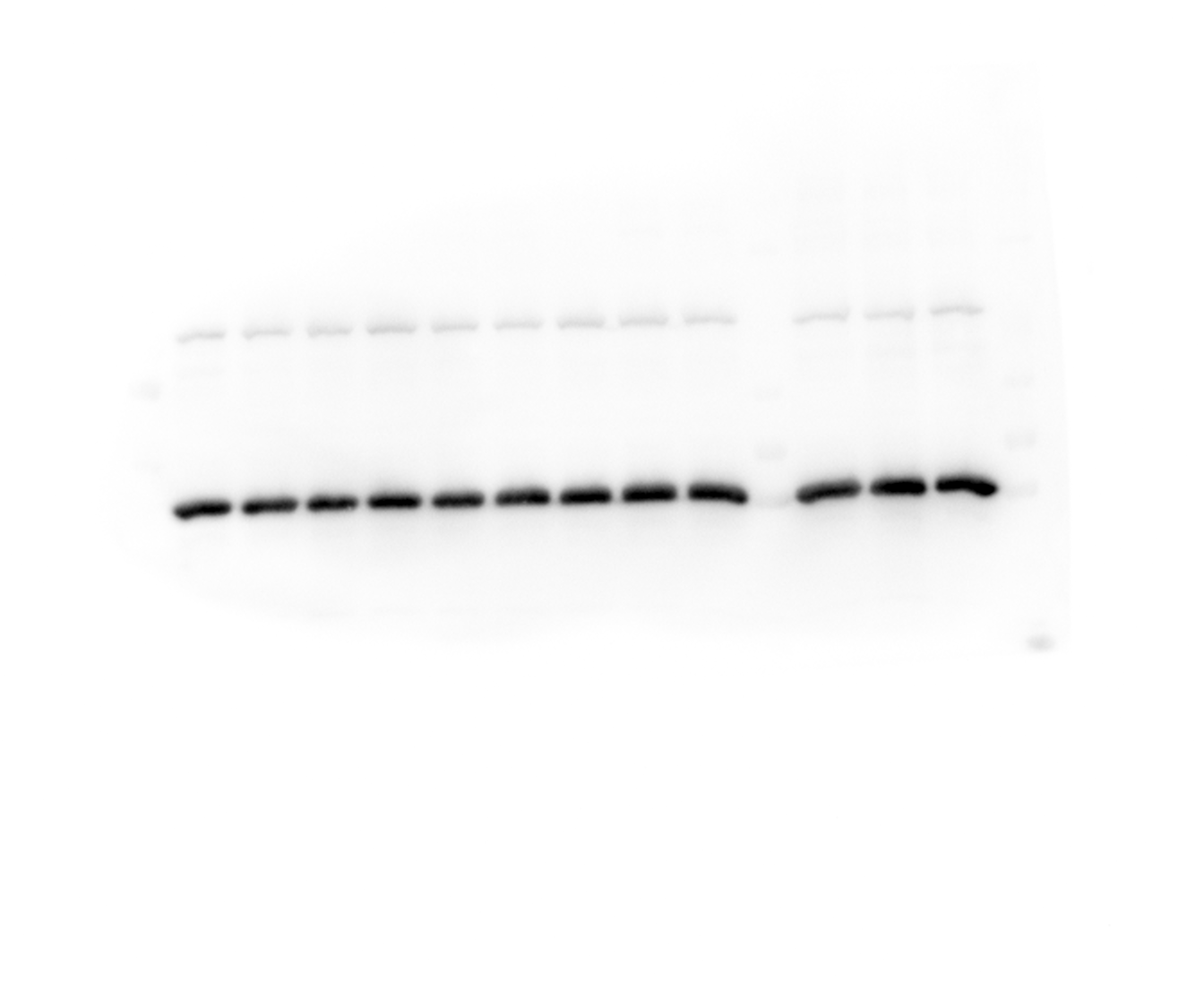

Supplement: Supplementary file 30 — Full and uncropped western blots [file 41420_2022_1171_MOESM30_ESM.tif]
