# Supplementary material for: PD-L1 enhances migration and invasion of trophoblasts by upregulating ARHGDIB via transcription factor PU.1
Source: Cell Death Discov. 2022 Sep 22;8:395. doi: 10.1038/s41420-022-01171-6 (PMC9500068; doi:10.1038/s41420-022-01171-6)
Supplement: Supplementary file 8 — Supplementary Legends [file 41420_2022_1171_MOESM8_ESM.docx]

Supplementary Figure Legends

Fig. S1. The RT–qPCR results validated the significant expression level changes of the most significant genes (ARHGDIB, PLXNA2, and SERPINA1), which are the target genes of PD-L1 identified by RNA-seq.

Fig. S2. The transcription factor PU.1 (SPI1) downstream of PD-L1 may affect the function of trophoblasts by regulating ARHGDIB. PU.1 (SPI1) belongs to the ETS family and is one of the proteins that are significantly upregulated in the PD-L1-overexpressing HTR-8/SVneo group.

Fig. S3. The DNA motif of PU.1 and the predicted binding sites on the ARHGDIB promoter were obtained from JASPAR, hTFtarget, and PROMO. Three sites where the transcription factor PU.1 is most likely to bind to the ARHGDIB promoter region (2000 bp).

Supplementary Table Legends

Supplementary Table 1. Western Blot Antibodies

Supplementary Table 2. Immunofluorescence Antibodies

Supplementary Table 3. PCR primer sequences
