# Supplementary material for: PD-L1 enhances migration and invasion of trophoblasts by upregulating ARHGDIB via transcription factor PU.1
Source: Cell Death Discov. 2022 Sep 22;8:395. doi: 10.1038/s41420-022-01171-6 (PMC9500068; doi:10.1038/s41420-022-01171-6)
Supplement: Supplementary file 31 — Western Blots merge PDF [file 41420_2022_1171_MOESM31_ESM.pdf]

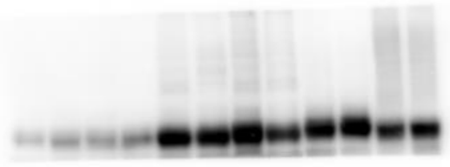

Fig1D PD-L1

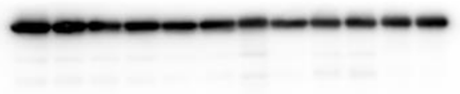

Fig1D GAPDH

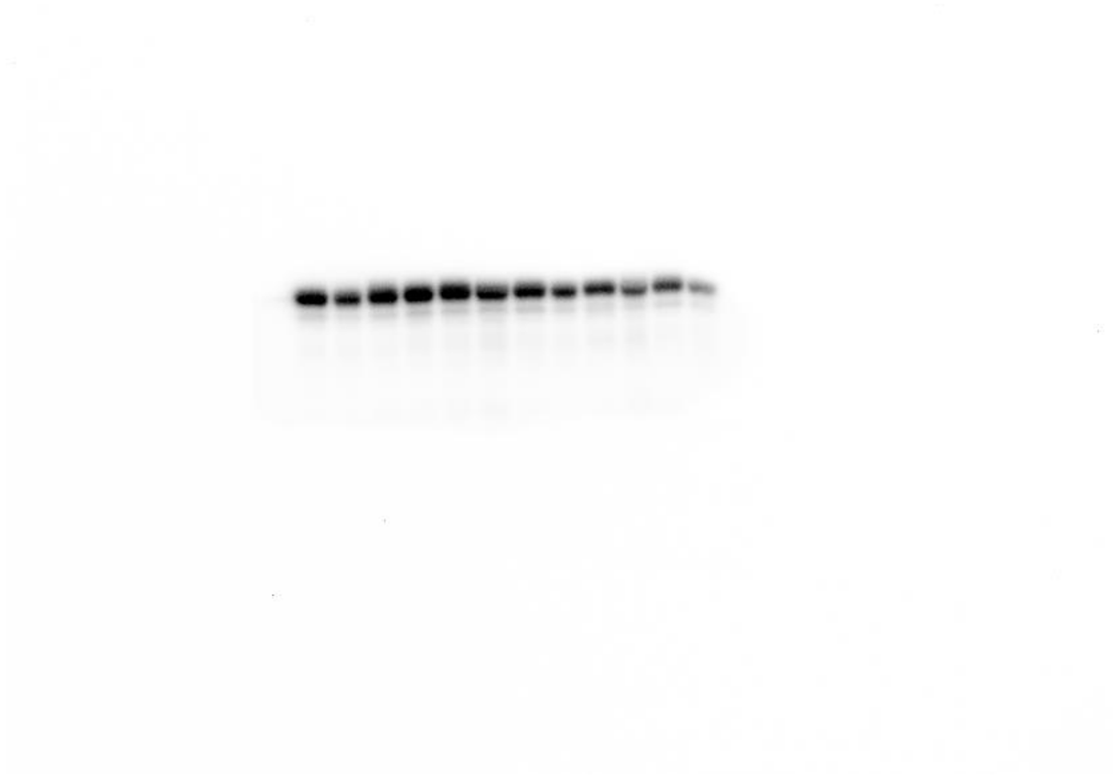

Fig 1E PD-L1

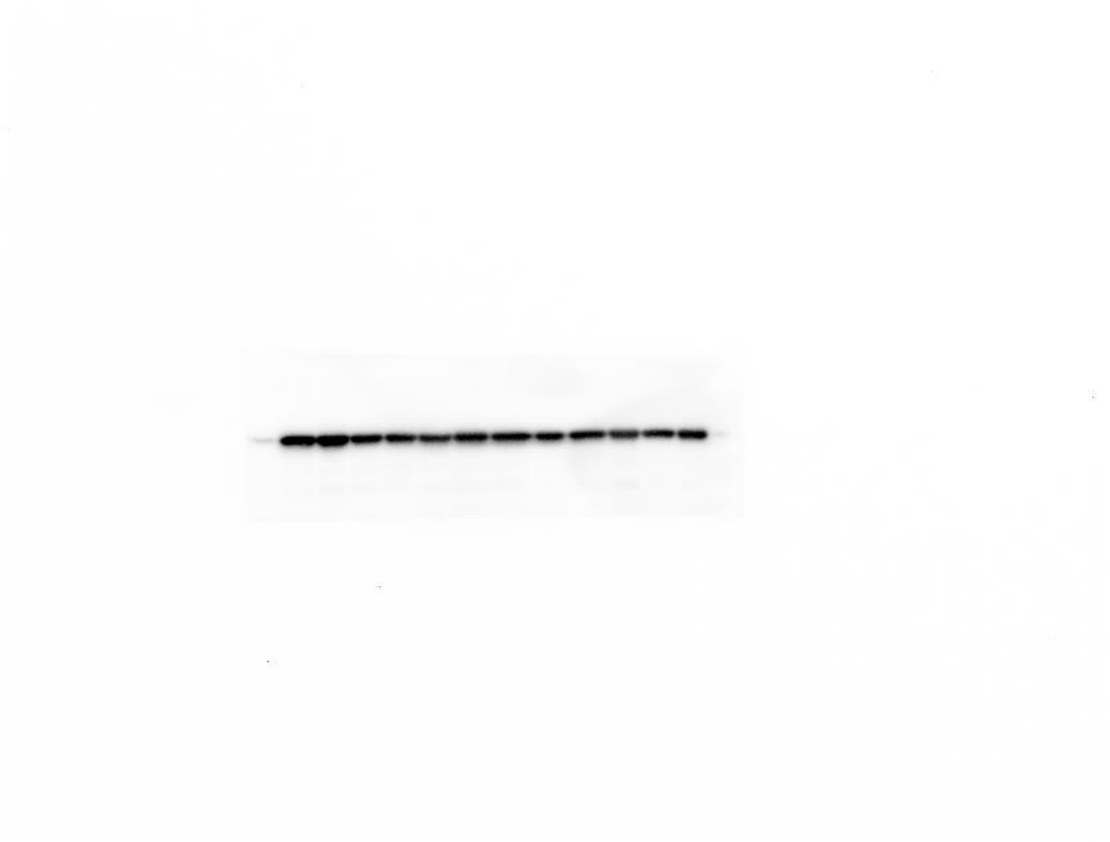

Fig 1E. GAPDH

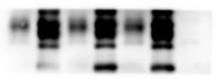

Fig 2A PD-L1

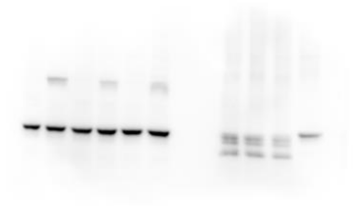

Fig 2A actin

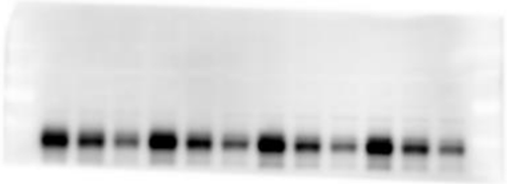

Fig 2B PD-L1

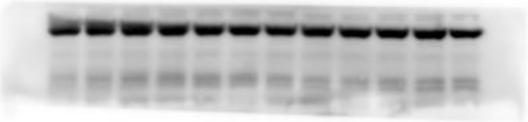

Fig 2B actin

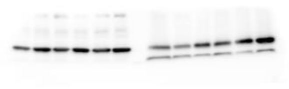

Fig 4E left ARHGDIB

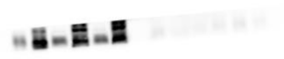

Fig 4E left PD-L1

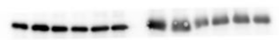

Fig 4E left actin

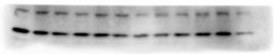

Fig 4E right ARHGDIB

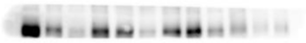

Fig 4E right PD-L1

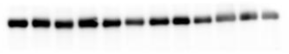

Fig 4E right actin

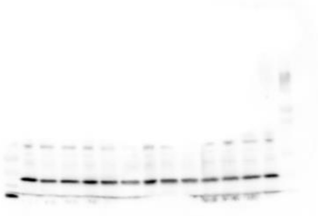

Fig 5A ARHGDIB

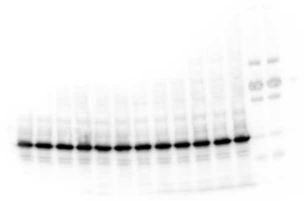

Fig 5A GAPDH

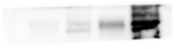

Fig 6B PD-L1

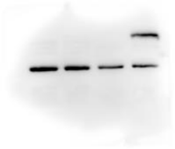

Fig 6B actin

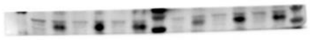

Fig 6D PU.1

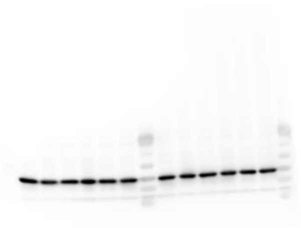

Fig 6D GAPDH

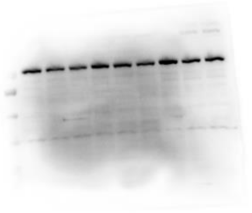

Fig 6E PU.1

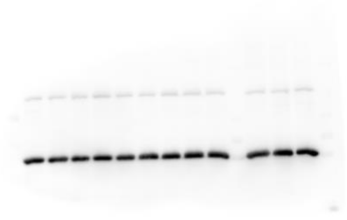

Fig 6E GAPDH
